# Single-cell and bulk transcriptome sequencing identifies two epithelial tumor cell states and refines the consensus molecular classification of colorectal cancer

**DOI:** 10.1038/s41588-022-01100-4

**Published:** 2022-06-30

**Authors:** Ignasius Joanito, Pratyaksha Wirapati, Nancy Zhao, Zahid Nawaz, Grace Yeo, Fiona Lee, Christine L. P. Eng, Dominique Camat Macalinao, Merve Kahraman, Harini Srinivasan, Vairavan Lakshmanan, Sara Verbandt, Petros Tsantoulis, Nicole Gunn, Prasanna Nori Venkatesh, Zhong Wee Poh, Rahul Nahar, Hsueh Ling Janice Oh, Jia Min Loo, Shumei Chia, Lih Feng Cheow, Elsie Cheruba, Michael Thomas Wong, Lindsay Kua, Clarinda Chua, Andy Nguyen, Justin Golovan, Anna Gan, Wan-Jun Lim, Yu Amanda Guo, Choon Kong Yap, Brenda Tay, Yourae Hong, Dawn Qingqing Chong, Aik-Yong Chok, Woong-Yang Park, Shuting Han, Mei Huan Chang, Isaac Seow-En, Cherylin Fu, Ronnie Mathew, Ee-Lin Toh, Lewis Z. Hong, Anders Jacobsen Skanderup, Ramanuj DasGupta, Chin-Ann Johnny Ong, Kiat Hon Lim, Emile K. W. Tan, Si-Lin Koo, Wei Qiang Leow, Sabine Tejpar, Shyam Prabhakar, Iain Beehuat Tan

**Affiliations:** 1 Genome Institute of Singapore, Agency for Science, Technology and Research (A*STAR), Singapore, Singapore; 2 Bioinformatics Core Facility, Swiss Institute of Bioinformatics, Lausanne, Switzerland; 3 National Cancer Centre, Singapore, Singapore; 4 Molecular Digestive Oncology, Department of Oncology, Katholieke Universiteit Leuven, Leuven, Belgium; 5 Hôpitaux Universitaires de Genève, Geneva, Switzerland; 6 University of Geneva, Geneva, Switzerland; 7 Duke-National University of Singapore Medical School, Singapore, Singapore; 8 MSD International GmbH (Singapore Branch), Singapore, Singapore; 9 National University of Singapore, Singapore, Singapore; 10 NantOmics, Rockville, MD USA; 11 Samsung Genome Institute, Samsung Medical Center, Seoul, Korea; 12 Department of Colorectal Surgery, Singapore General Hospital, Singapore, Singapore; 13 EL Toh Colorectal & Minimally Invasive Surgery, Singapore, Singapore; 14 Department of Sarcoma, Peritoneal and Rare Tumours (SPRinT), Division of Surgery and Surgical Oncology, National Cancer Centre Singapore, Singapore, Singapore; 15 Department of Sarcoma, Peritoneal and Rare Tumours (SPRinT), Division of Surgery and Surgical Oncology, Singapore General Hospital, Singapore, Singapore; 16 Laboratory of Applied Human Genetics, Division of Medical Sciences, National Cancer Centre Singapore, Singapore, Singapore; 17 SingHealth Duke-NUS Oncology Academic Clinical Program, Duke-NUS Medical School, Singapore, Singapore; 18 SingHealth Duke-NUS Surgery Academic Clinical Program, Duke-NUS Medical School, Singapore, Singapore; 19 Institute of Molecular and Cell Biology, A*STAR Research Entities, Singapore, Singapore; 20 Department of Anatomical Pathology, Singapore General Hospital, Singapore, Singapore

**Keywords:** Colorectal cancer, Transcriptomics, Gene expression profiling

## Abstract

The consensus molecular subtype (CMS) classification of colorectal cancer is based on bulk transcriptomics. The underlying epithelial cell diversity remains unclear. We analyzed 373,058 single-cell transcriptomes from 63 patients, focusing on 49,155 epithelial cells. We identified a pervasive genetic and transcriptomic dichotomy of malignant cells, based on distinct gene expression, DNA copy number and gene regulatory network. We recapitulated these subtypes in bulk transcriptomes from 3,614 patients. The two intrinsic subtypes, iCMS2 and iCMS3, refine CMS. iCMS3 comprises microsatellite unstable (MSI-H) cancers and one-third of microsatellite-stable (MSS) tumors. iCMS3 MSS cancers are transcriptomically more similar to MSI-H cancers than to other MSS cancers. CMS4 cancers had either iCMS2 or iCMS3 epithelium; the latter had the worst prognosis. We defined the intrinsic epithelial axis of colorectal cancer and propose a refined ‘IMF’ classification with five subtypes, combining intrinsic epithelial subtype (I), microsatellite instability status (M) and fibrosis (F).

## Abstract (web summary)

A single-cell transcriptomic analysis of 63 patients with colorectal cancer classifies tumor cells into two epithelial subtypes. An improved tumor classification based on epithelial subtype, microsatellite stability and fibrosis reveals differences in pathway activation and metastasis.

## Main

Colorectal cancer (CRC) is a heterogeneous disease. In 2015, based on gene expression profiles from bulk tumors, an international consortium identified four CMSs (CMS1–4)^1^, characterized respectively by enriched features of immune infiltration, canonical WNT and Myc activation, metabolic dysregulation and a mesenchymal fibrotic reaction. The CMS subtypes have been reproduced across multiple studies and are thought to represent four distinct subtypes^2,3^. The fibrotic CMS4 subtype portends poor relapse-free survival (RFS)^1,4^.

Bulk transcriptomes measure total gene expression in heterogeneous tissues, and hence transcriptomes of component cells, their proportions and tumor microenvironment interactions are obscured. Cell-type admixture confounds computational deconvolution^5,6^ of cell-type-specific gene expression. Profiling patient-derived xenografts, wherein human stromal cells are depleted, led to the CRC intrinsic subtypes (CRIS) classification^7^. However, this is limited by cell-type contamination, bulk expression effects and experimental artefacts of human tumors propagated across species.

Single-cell RNA sequencing (scRNA-seq) characterizes transcriptomes at cellular resolution, enabling identification of cell types and their expression profiles. As a proof-of-principle, we identified two major cancer-associated fibroblast subtypes (CAF-A, CAF-B) in the first CRC scRNA-seq analysis^8^. We subsequently generated an scRNA-seq atlas of 91,103 cells from 29 patients, providing a global cellular landscape of CRC^9^.

**Fig. 1:**
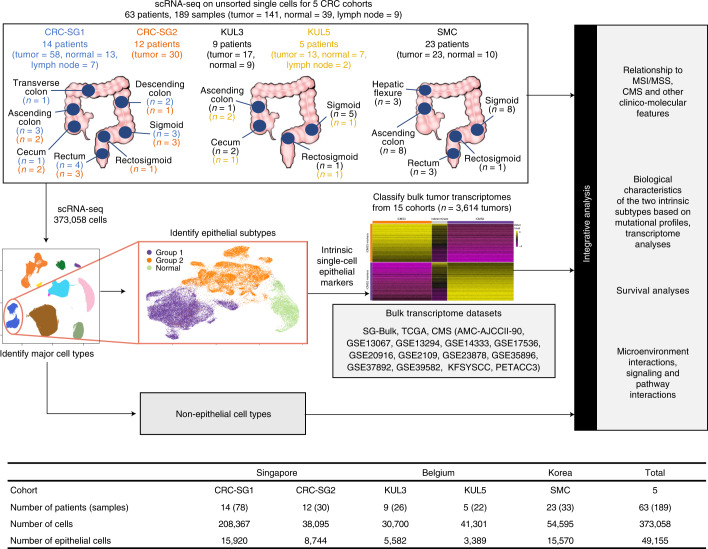
Study schema. For each of the five cohorts, the number of patients, anatomical locations and the number of samples profiled using scRNA-seq are indicated at the top. The major stages of data analysis are indicated below and to the right. For each cohort, the total number of profiled cells and the number of epithelial cells are indicated at the bottom. Single-cell transcriptomes from the SMC cohort and six patients from KUL3 (75,332 cells in all after QC) have been previously reported^9^.

Here, we sought to study the epithelial subtypes that underpin the molecular classification of CRC. Single-cell transcriptomes of 141 tumor samples, 39 adjacent normal samples and 9 lymph node samples from 63 patients across five cohorts were integrated to construct one of the largest single-cell CRC datasets to date (Fig. 1). Of the 373,058 single cells profiled, we focused primarily on the 49,155 epithelial cells (Fig. 1). Remarkably, amongst malignant epithelial cells, two distinct subtypes consistently emerged after independent analyses of single-cell expression, regulon and inferred copy number profiles, suggesting a common genetic program dictating two major epithelial subtypes in CRC. We quantified our intrinsic epithelial signature in 3,614 bulk transcriptomes across 15 datasets and recapitulated these two intrinsic subtypes. We observed a correspondence to the CMS classification and termed the two epithelial groups intrinsic-consensus molecular subtypes (iCMSs), consisting of iCMS2 (i2) and iCMS3 (i3). Amongst MSS cancers, most CMS2 and CMS3 tumors had iCMS2 and iCMS3 epithelium, respectively. MSI-H and CMS1 cancers were generally classified as iCMS3. Importantly, we found that MSS tumors with iCMS3 epithelium (iCMS3_MSS) had transcriptomic, genomic and biological pathway enrichment features that were more similar to MSI-H cancers than to iCMS2_MSS cancers. The fibrotic CMS4 group comprised cancers with either iCMS2 or iCMS3 epithelial cells, suggesting that fibrosis is orthogonal to the intrinsic CRC epithelial structure. Importantly, while CMS4 as a whole is associated with poor RFS, we identified the subclass of fibrotic CMS4 cancers with iCMS3 epithelial cells that had the worst prognosis found. With these insights, we propose a refinement of the classification of CRC, the IMF classification, comprising intrinsic epithelial subtypes, microsatellite instability status and fibrosis.

## Results

### Cell-type annotation

**Fig. 2:**
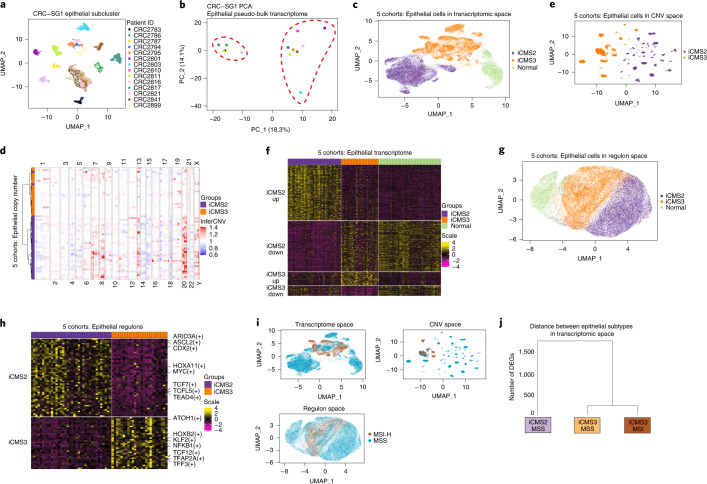
The discovery of iCMS subtype in scRNA-seq. **a**, Reduced-dimensionality (UMAP) visualization of epithelial single cells (*n* = 15,920) in transcriptome space: 14 patients from CRC-SG1, colored by patient ID. **b**, Same dataset, PCA visualization of 14 patient-specific epithelial pseudo-bulk transcriptomes. **c**, UMAP visualization of 49,155 epithelial cells from five cohorts in transcriptomic space colored by iCMS subtype. **d**, Heatmap of 63 patient-specific pseudo-bulk-inferred CNV scores. Columns were sorted based on their chromosomal position while rows were clustered using hierarchical clustering. **e**, UMAP visualization of 49,155 epithelial cells from five cohorts in CNV space with each cell represented by its vector of inferred copy number scores in genomic bins. **f**, Heatmap of expression of 715 DEGs in patient-specific pseudo-bulk transcriptomes. Only the 61 patients with consistent iCMS classification were used. Each gene is zero-centered and scaled to unit variance. **g**, UMAP visualization of 46,006 epithelial cells from 61 patients in regulon space (SCENIC analysis). **h**, Patient-specific pseudo-bulk heatmap of 90 differentially expressed regulons: 61 patients, colored by scaled regulon activity score (AUC score). **i**, UMAP plot of epithelial cells in transcriptomic, copy number and regulon space, colored by MSI status. **j**, Dendrogram showing the distance between epithelial subtypes in transcriptomic space. The number of DEGs was defined as the pairwise distance and the matrix of pairwise distances was used for tree construction. AUC, Area Under Curve; UMAP, Uniform Manifold Approximation and Projection.

**Extended Data Fig. 1:**
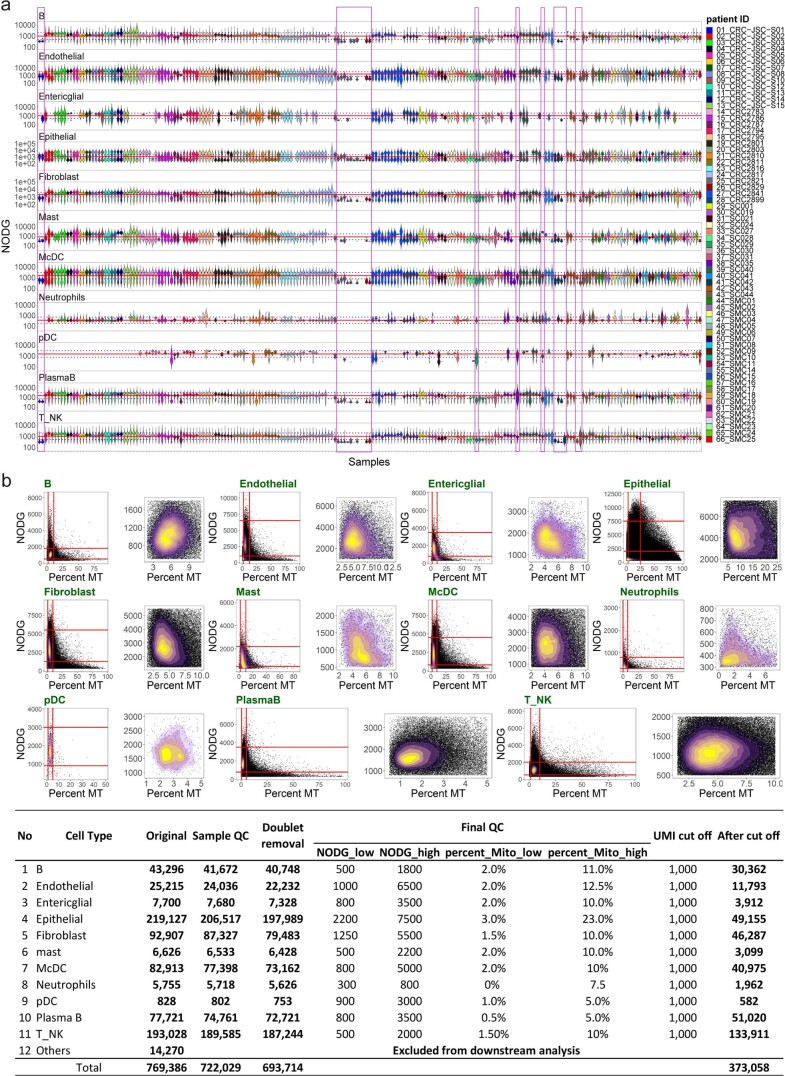
QC cut off for all cells in 5 cohorts. Violin plot showing number of detected genes (NODG) distribution of each sample in their respective cell types. Solid red lines indicate median NODG across 211 samples in each respective cell type, while dashed blue lines indicate 2 or 0.5 times the red line. Magenta box highlight samples with median NODG consistently higher or lower than the dashed blue lines in more than half of the cell types, which were discarded from this study (22 samples from 9 patients). b. Density plot and numeric table for all cell types in 5 cohorts before and after quality control (QC). Red lines in the density plots indicate the QC parameter that was used. The 11 major cell types are: B cells (B), Endothelial cells, Epithelial cells, Fibroblast cells, Granulocyte cells, monocyte conventional dendritic cell (McDC), Plasma-B cells, T and NK cells (T_NK).

**Extended Data Fig. 2:**
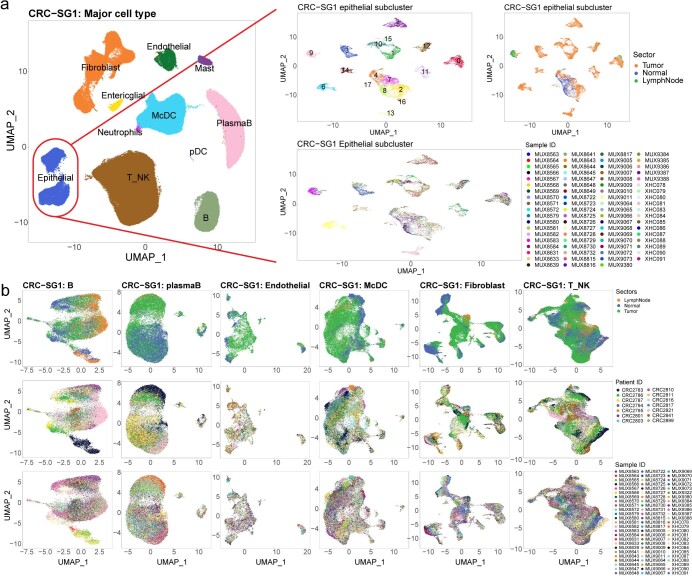
Sub-clustering of all major cell type in CRC-SG1 cohorts. **a**. (Left) UMAP visualization of clusters representing major cell types in CRC-SG1 (n = 208,367), (Right) Epithelial sub-cluster colored by cluster ID, tumor sectors, and sample ID (n = 15,920). **b**. UMAP visualization of B (n = 19,088 cells), Plasma-B (n = 26,710 cells), Endothelial (n = 6,875 cells), monocyte conventional dendritic cell (McDC) (n = 26,127 cells), Fibroblast (n = 31,415 cells), and T and NK (T_NK) (n = 76,812 cells) subclusters from 14 patients in CRC-SG1 dataset, colored by tumor sectors (upper panel), patient ID (middle panel), and sample ID (lower panel).

scRNA-seq data were filtered to discard low-quality cells and doublets (Supplementary Fig. 1, Extended Data Fig. 1 and Methods). Supervised clustering (Reference Component Analysis v2 (RCA2)) at low resolution grouped cells into 11 major cell types (Extended Data Fig. 1). To identify epithelial cell subtypes, we initially analyzed the largest cohort, ‘CRC-SG1’ (Singapore, 208,367 cells, 15,920 epithelial), with multiple tumor sectors per patient, serving as biological replicates (Fig. 1 and Extended Data Fig. 1). We performed de novo clustering on CRC-SG1 epithelial cells using DUBStepR^10^ for feature selection, and then re-clustered the cells using differentially expressed genes (DEGs) between the initial clusters (Supplementary Fig. 1 and Methods). One cluster comprised all cells from normal samples and a minority of cells from tumor samples (23.4%). These represent normal cells in tumor sectors. Malignant cells from tumor samples formed patient-specific clusters, each of which included cells from different sectors of the same tumor (biological replicates) (Fig. 2a and Extended Data Fig. 2a). All other major cell types showed minimal patient specificity (Extended Data Fig. 2b). Thus, patient-specific transcriptomic clusters formed by tumor epithelial cells likely represent biological differences between patients, rather than batch effects.

Principal component analysis (PCA) demarcated two distinct epithelial subgroups (Fig. 2b). We used 848 DEGs (Supplementary Fig. 2a) between these two groups to cluster 33,235 epithelial single-cell transcriptomes from the four other cohorts (111 samples, 49 patients) (Supplementary Fig. 2b and Methods). In all four cohorts, we again observed coclustering of normal cells and numerous patient-specific malignant cell clusters that formed two distinct groups in PCA (Supplementary Fig. 2b,c). We then combined the five cohorts (189 samples, 63 patients) and used the 848 DEGs to cluster epithelial cells, and again recovered two major tumor subtypes (iCMS2 and iCMS3; Fig. 2c).

We noticed that genes upregulated in iCMS2 epithelial cells relative to iCMS3 and normal-like epithelium in CRC-SG1 were enriched in specific chromosomal arm gains, including 8q, 13q and 20q (Supplementary Fig. 2d). We therefore used inferCNV^11^ to infer copy number variants (CNVs) from epithelial cell transcriptomes of all five cohorts and observed that, despite substantial patient-to-patient variability within iCMS2, 7pq, 8q, 13q and 20pq were frequently gained and 1p, 4pq, 8p, 14q, 15q, 17p and 18pq were frequently deleted (Fig. 2d). In contrast, iCMS3 tumors were diploid or showed infrequent and inconsistent copy number alterations. Moreover, patients were clustered by their pseudo-bulk copy number profiles, and we recapitulated the iCMS2–iCMS3 dichotomy observed in epithelial single-cell transcriptomes, with only 2 of 63 patients (3%) showing discordant grouping (Fig. 2d). The separation of i2 and i3 tumors was observed even when epithelial single cells from the five cohorts were visualized in inferred copy number space (Fig. 2e), suggesting copy number alterations contribute to the observed dichotomy in CRC epithelial transcriptomes. We performed differential expression analysis on epithelial pseudo-bulk transcriptomes from the 61 consistent patients to define an intrinsic epithelial cancer signature comprising 715 genes (Fig. 2f, Supplementary Fig. 2e and Methods).

To evaluate the relationship of the two single-cell-defined intrinsic epithelial groups to CMS groups identified by bulk gene expression, we first identified marker genes for each CMS subtype that also showed epithelial-specific expression in our five scRNA-seq datasets. We then used these four gene sets to construct four epithelial CMS metagene expression scores for each malignant cell. Upregulation of the CMS2 epithelial metagene was the most prominent feature of iCMS2 cells. Similarly, upregulation of the CMS3 epithelial metagene distinguished iCMS3 cells (Supplementary Fig. 3). Thus, our single-cell iCMS classification may represent the core epithelial intrinsic components of bulk CMS.

Next, we used SCENIC^12^ to infer single-cell activity scores for the regulons of 347 transcription factors and used these scores to cluster epithelial cells from the 61 patients. The same two intrinsic epithelial subtypes again self-emerged, with 90 differential regulons including TCF/LEF (T cell factor/lymphoid enhancer factor family), MYC and homeobox transcription factors, suggesting a pervasive gene regulatory program underpinning the biology of these two epithelial subtypes (Fig. 2g,h and Supplementary Fig. 4).

**Extended Data Fig. 3:**
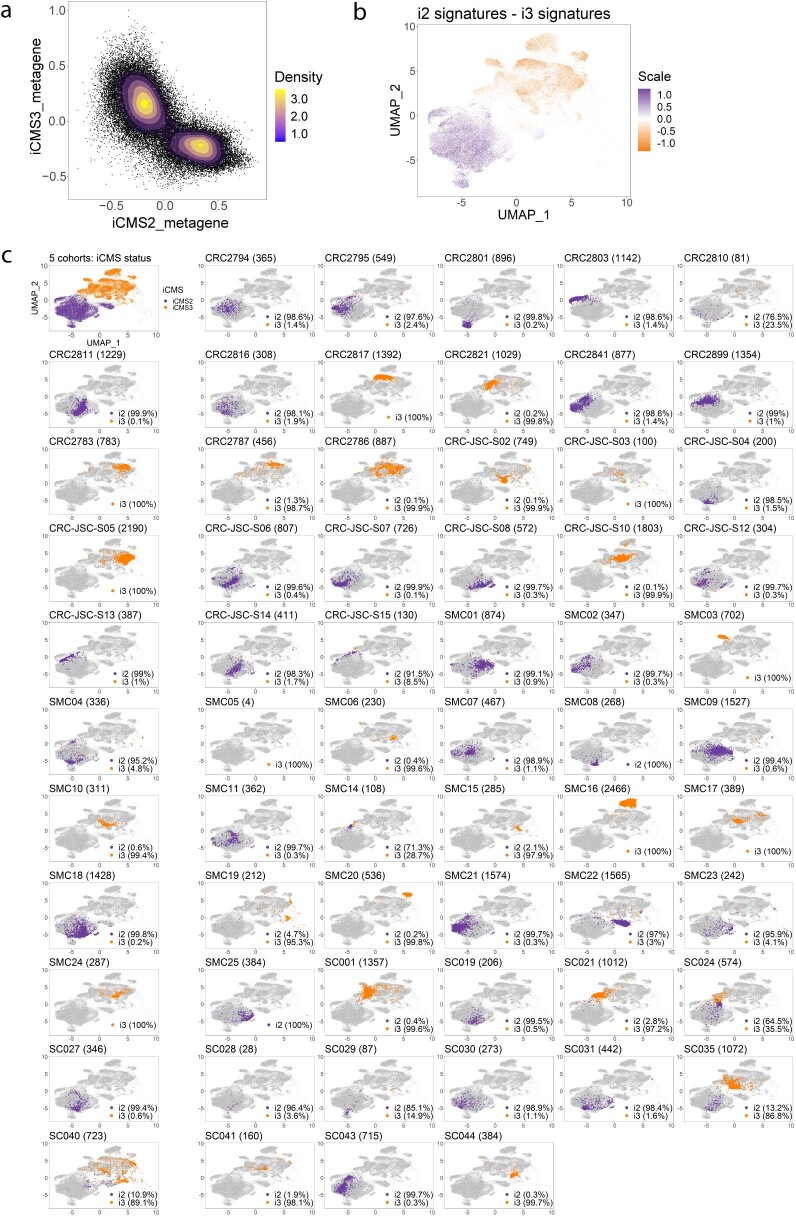
The bimodality of iCMS subtypes. **a**. Density plot of 42,010 tumor epithelial cells from 5 cohorts in iCMS metagene space. The mode at the bottom right of the scatterplot corresponds to iCMS2 cells, while the opposite mode corresponds to iCMS3. **b**. UMAP visualization of 42,010 tumor epithelial cells from 5 cohorts in transcriptomic space colored by iCMS score for each individual cell, defined as (iCMS2 metagene expression score) - (iCMS3 metagene expression score). **c**. UMAP visualization of tumor epithelial cells from 5 cohorts in transcriptomic space, grouped by patients, and colored by iCMS label. The numbers next to patient ID in each plot indicates the total number of tumor epithelial cells for that particular patient. The percentage on the bottom right of the UMAP indicates the number of cells that were clustered in i2 clusters (purple color) or i3 clusters (orange color).

To characterize the distribution of malignant cells as distinct transcriptomic states (versus a continuum), we calculated their i2 and i3 metagene expression scores by averaging the 715 iCMS marker genes (Fig. 2f) within the same iCMS group (Supplementary Notes). The metagene score distribution was bimodal, with one mode corresponding to i2-like transcriptomes and the other i3-like (Extended Data Fig. 3a,b and Supplementary Notes), supporting distinct i2 and i3 epithelial cell states. By cluster label, >95% of malignant cells belonged to a single cluster (either i2 or i3) (Extended Data Fig. 3c). By i2 and i3 metagene score, >80% of cells were either preferentially i2 or preferentially i3 (score difference > 0.1) in 54 of 63 tumors (86%) (Supplementary Fig. 5). Thus, in most tumors, the large majority of cells belong to a single iCMS type, with hybrid tumors being infrequent.

### Transcriptomic distances

Across transcriptomic, CNV and regulon spaces (Fig. 2i), a group of MSS epithelial cells comingled with MSI-H cells within the i3 cluster, suggesting biological programs in i3_MSS are more similar to MSI-H than to i2_MSS. Using the number of pseudo-bulk DEGs as a distance measure, we constructed a dendrogram to represent the relationships between these malignant cell groups. Consistently, i3_MSS has much greater similarity to i3-MSI-H than to i2_MSS (Fig. 2j).

### Epithelial subtypes in an independent scRNA-seq dataset

We re-analyzed single-cell transcriptomes from a recent study of 62 patients with CRC^13^, which had focused on cell-type differences between MSI-H and MSS tumors. Using the above-described quality control (QC) cutoffs, we identified 56,551 high-quality epithelial cells and clustered them using the 715 iCMS marker genes, again identifying three clusters: normal, i2 and i3 (Supplementary Fig. 6a). Once again, cells from i3_MSS and MSI-H tumors intermingled within the i3 cluster, while cells from i2_MSS tumors formed a distinct i2 cluster. Similarly, i2 and i3 metagene scores showed clear bimodality. In >90% of patients, >90% of malignant cells belonged to a single subtype (Supplementary Fig. 6b). These results corroborate two intrinsic transcriptomic subtypes, iCMS2 and iCMS3, with i3-MSS and MSI-H malignant cells being highly similar.

### Classification of bulk transcriptomes

**Fig. 3:**
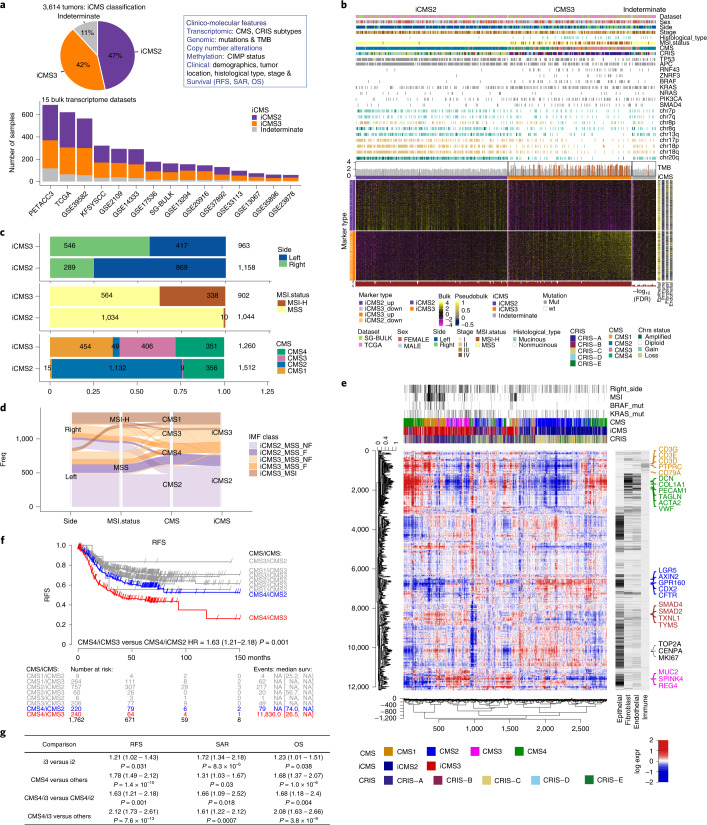
iCMS classification of bulk transcriptomes. **a**, Proportion of 3,614 patients classified as iCMS2, iCMS3 or indeterminate based on their bulk tumor transcriptome. The box on the right lists the parameters that will be correlated with iCMS, including: CMS, CRIS, CIMP, TMB and copy number variation, overall survival (OS), survival after relapse (SAR) and RFS. **b**, Heatmap of 715 iCMS marker genes used to classify the 455 TCGA and SG-Bulk tumor transcriptomes. Gene expression values were log-transformed, zero-centered and scaled to unit variance. Upper annotation bars show clinical, mutational and copy number gain/loss categorized as amplified (≥4 copies), gain (2.5–4 copies), diploid (1.5–2.5 copies) and loss (<1.5 copies), as well as TMB (MSI-H patients highlighted in brown). Right annotation bar shows the average scaled expression of each gene across four major cell types, based on scRNA-seq data from the CRC-SG1 cohort. Lower annotation track: FDR *Q* value of iCMS classification. **c**, Breakdown of iCMS2 and iCMS3 samples by anatomical side (top), MSI status (middle) and CMS (bottom). Statistics are based on all bulk tumor datasets, including only those for which the relevant annotations are available. **d**, Bulk tumor datasets: alluvial plot demonstrating the relationship between IMF classification and anatomical side, MSI status, CMS subtype and iCMS. **e**, Heatmap showing the coexpression pattern of 2,873 bulk tumor transcriptomes from 14 clinical cohorts. Rows are genes; columns are patients; ordering is by unsupervised hierarchical clustering. Gene expression values are normalized as in **b**. CMS, iCMS and CRIS labels are indicated above the map, together with selected clinical parameters. Annotation bars for four major tumor cell types are as in **b. f**, Kaplan–Meier plot of RFS of patients classified by CMS and iCMS. The table below the graph indicates the number of patients at risk for all groups at various time points, followed by the number of events and median survival (in months) with their confidence intervals. **g**, Summary table of survival analysis conducted in this study. *P* values are Cox proportional hazard models (as implemented by R survival package). FDR, False Discovery Rate.

**Extended Data Fig. 4:**
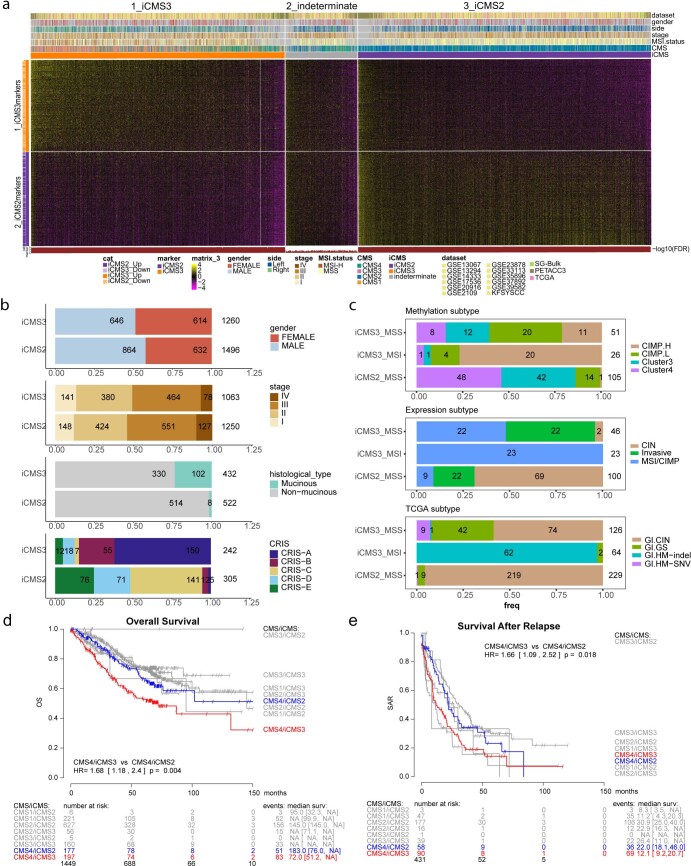
iCMS classification in 15 bulk datasets. **a**. Heatmap of 715 iCMS genes used to classify 3,614 samples across 15 datasets by NTP, colored by scaled gene expression, arranged by sum of expression of signature genes. Top annotation bars show clinical information for each sample (dataset, gender, side, stage, MSI status, CMS, iCMS). Bottom annotation shows the FDR of NTP classification. **b**. Proportions of gender, stage, histological type and CRIS subgroup from 15 bulk datasets; number of samples in each group is labelled. For each analysis, only samples with information available were used. **c**. Proportions of methylation subtype (n = 182, top, as defined in the original paper^1^), expression subtype (n = 169, middle) and TCGA subtype (n = 419, bottom) in iCMS2_MSS, iCMS3_MSS and iCMS3_MSI from TCGA; number of samples in each group is labelled. (**d,e**) Kaplan-Meier plot of overall survival (**d**) and survival after relapse (**e**) for all patients classified by CMS and iCMS.

We used the 715 iCMS marker genes to classify 3,614 tumor bulk transcriptomes from 15 primary tumor datasets (The Cancer Genome Atlas (TCGA), SG-Bulk and 13 CMS cohort datasets) and similarly observed two groups with either high i2 or high i3 signatures (Fig. 3a and Extended Data Fig. 4a). Using nearest template prediction, 47% of the tumors were classified as i2 and 42% as i3 at *Q* value < 0.05. Most tumors were robustly classified by nearest template prediction even at *Q* value < 0.005. i2 and i3 tumors were identified at relatively similar proportions across multiple datasets and i2/i3 tumors from different datasets comingled (Fig. 3b and Extended Data Fig. 4a). This indicates that the intrinsic epithelial signatures can robustly identify the epithelial subtypes from bulk tumor transcriptomes.

### Relationship with CMS and clinico-molecular characteristics

We examined clinical and molecular features of iCMS subtypes (Fig. 3a–c and Extended Data Fig. 4a–c). As before, we found that almost all MSI-H tumors were classified as iCMS3, together with the subgroup of iCMS3_MSS tumors. DNA methylation was a feature of the MSI-H group, although CpG island methylation phenotype (CIMP) status did not neatly substratify the MSS groups (Extended Data Fig. 4c). Right-sided tumors were mainly i3 (66%), and left-sided tumors mainly i2 (68%). Mucinous cancers of both MSI-H and MSS subgroups were mainly i3 (93%).

CMS1 (97%) and CMS3 (98%) tumors were mainly i3, while CMS2 (96%) tumors were mainly i2. However, CMS4 tumors can be either i2 or i3 (with an equal proportion), suggesting that fibrosis is decoupled from intrinsic epithelial structure (Fig. 3c,d).

We performed hierarchical clustering on 2,873 bulk tumor transcriptomes from 14 clinical cohorts. For each gene, we related the bulk expression to its expression in our single-cell dataset, stratified by major cell-type cohorts (Fig. 3e). We observed that tumors grouped together based on iCMS, MSI status and bulk CMS. At the highest level, iCMS2 and iCMS3 tumors separated, presumably due to distinct epithelial transcriptomes. Within iCMS3, MSI-H tumors mostly segregated as a subcluster characterized by high expression of immune-specific genes and minimal expression of fibroblast-specific genes. Within each of the major iCMS2 and iCMS3 groups, we observed a subset of fibrotic (CMS4) tumors characterized by increased expression of fibroblast, endothelial and some immune-specific genes. This suggests that iCMS, MSI status and CMS jointly inform the molecular classification of CRC. Tumors were not organized by CRIS–epithelial subgroups (Fig. 3e and Extended Data Fig. 4b).

### Survival analysis

Across 1,762 tumors with survival data (Fig. 3f,g), CMS4 showed poor Relapse Free Survival (RFS) (hazard ratio (HR) = 1.78, *P* = 1.4 × 10^−10^), consistent with the literature^1^. Poor RFS was a particular feature of the CMS4/iCMS3 subgroup (Fig. 3f) (CMS4/iCMS3 versus all others: HR = 2.12, *P* = 7.6 × 10^−13^; CMS4/iCMS3 versus CMS4/iCMS2: HR = 1.63, *P* = 0.001). This effect also extended to inferior overall survival (Extended Data Fig. 4d; CMS4/iCMS3 versus all others: HR = 2.08, *P* = 3.8 × 10^−9^; CMS4/iCMS3 versus CMS4/iCMS2: HR = 1.68, *P* = 0.004). Survival after relapse was worse for i3 cancers relative to i2 (HR = 1.72, *P* = 8.3 × 10^−6^), as was overall survival (HR = 1.23, *P* = 0.04) (Extended Data Fig. 4e).

### IMF classification

Microsatellite instability marks a subset of i3 cancers. Fibrotic CMS4 cancers were stratified by epithelial subtype (i2 versus i3) into two subgroups with distinct microenvironment composition and distinct likelihood of survival. Putting these together, we propose a refinement of the four-group bulk CMS classification, based on the three biological layers of intrinsic epithelial status (I), microsatellite status (M) and presence of fibrosis (F), termed ‘IMF’. IMF stratifies tumors into five commonly occurring classes: iCMS2_MSS_NF, iCMS2_MSS_F, iCMS3_MSS_NF, iCMS3_MSS_F and iCMS3_MSI.

### Genomic features and functional associations

**Fig. 4:**
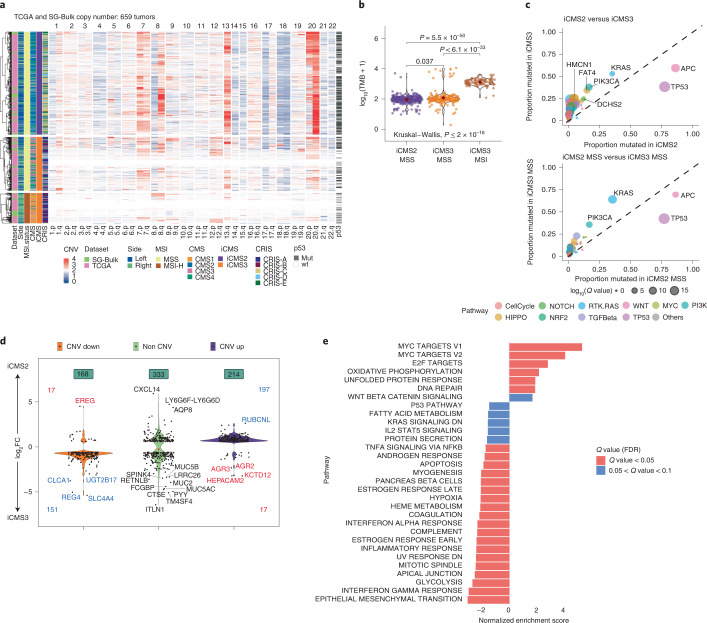
Relationship of iCMS to genomic features. **a**, Copy number variation by chromosome arm in 659 patients from TCGA and SG-Bulk cohorts. Samples are ordered as i2_MSS, i3_MSS, i3_MSI. p53 mutation status is shown on the right for each sample. **b**, TMB in iCMS2_MSS (*n* = 389), iCMS3_MSS (*n* = 195) and iCMS3_MSI (*n* = 116) samples from TCGA and SG-Bulk data. Pairwise *P* values: two-sided Wilcoxon rank-sum test; overall *P* value: Kruskal–Wallis test. **c**, Scatterplot of proportion of TCGA and SG-Bulk samples with mutations in 333 CRC-associated genes, in iCMS2 (*n* = 344) versus iCMS3 (*n* = 281) (top) and iCMS2_MSS (*n* = 338) versus iCMS3_MSS (*n* = 181) (bottom). Dot size corresponds to *Q* value by two-sided Fisher’s exact test with Benjamini–Hochberg correction. Only genes with *Q* value < 0.05 and proportion mutated > 0.2 are labeled. **d**, Violin plot showing the expression fold-change of 715 iCMS marker genes in i2 relative to i3, categorized by copy number status. CNV Up, DEGs on chromosomal arms with frequent increase in copy number in i2; CNV Down, DEGs on arms with frequent loss of copy number in i2; red font, DEGs whose expression fold-change is discordant with the copy number change; blue font, concordant. **e**, GSEA results of MSigDB hallmark pathways in iCMS2 versus iCMS3. *X* axis, normalized enrichment score in iCMS2 relative to iCMS3. FC, fold-change.

**Extended Data Fig. 5:**
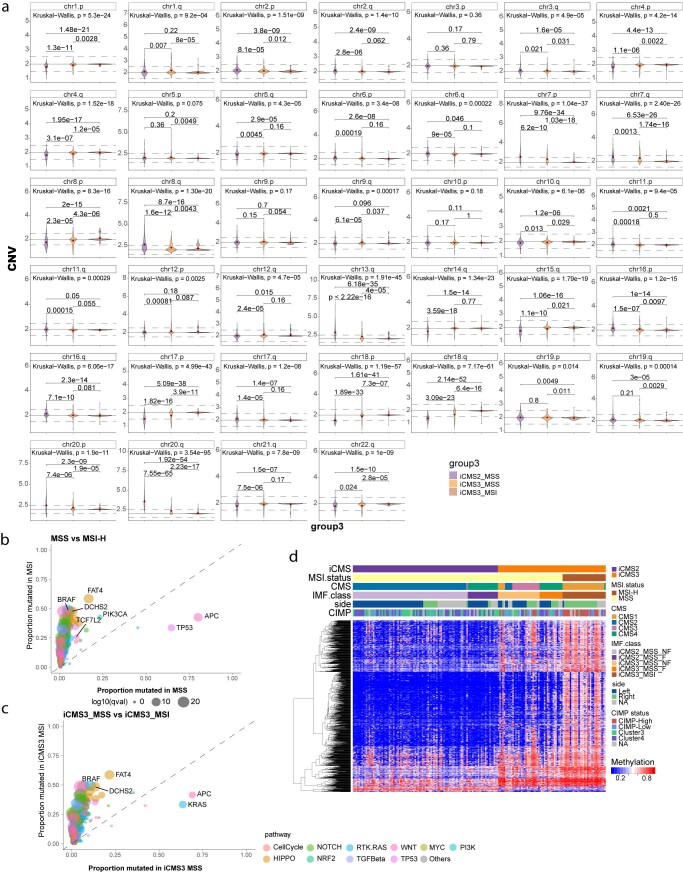
Genomic features in iCMS, MSI, and CIMP status. **a**. Log2 copy-number ratios for each chromosome arm in iCMS2_MSS (n = 363), iCMS3_MSS (n = 189) and iCMS3_MSI (n = 107) from TCGA and SG-Bulk datasets. P-values for pairwise comparisons are by two-sided Wilcoxon rank-sum test; overall p-value is by Kruskal-Wallis test. Scatterplot of proportion of TCGA and SG-Bulk samples with mutations in 333 CRC-associated genes, in **b**. MSS (n = 519) vs MSI (n = 101) and **c**. iCMS3_MSS (n = 181) vs iCMS3_MSI (n = 99) (right). Dot size corresponds to q-value by Fisher’s exact test with Benjamini-Hochberg correction. Only genes with q-value <0.05 and proportion mutated > 0.5 are labelled. (**d**) Heatmap of differentially methylated CpG sites (n = 978) between iCMS2 and iCMS3 subtypes in 176 TCGA samples.

We examined the copy number architecture of iCMS3_MSI, iCMS3_MSS and iCMS2_MSS tumors based on 659 tumors from the TCGA and SG-Bulk cohorts (Fig. 4a). i2 tumors were driven by copy number changes in specific chromosomes commonly altered in CRC^14,15^. Gains in 7pq, 8q, 13q and 20pq and losses of 1p, 4pq, 8p, 14q, 15q, 17p and 18pq characterized i2 tumors. i3 tumors were either MSI-H and diploid or MSS tumors with fewer copy number changes than i2 (Fig. 4a and Extended Data Fig. 5a). *TP53* mutations were more prevalent in i2 MSS tumors than in i3, perhaps contributing to overall genomic instability in the former group (Fig. 4a,c). This pattern of CNV enrichment is consistent with our inferCNV^11^ analysis of single cells from i2 and i3 tumors (Fig. 2d).

Tumor mutation burden (TMB) was higher among i3_MSI tumors (median: 1,353) and similar between i3 and i2 MSS tumors (median: 98 and 105 for i2_MSS and i3_MSS, respectively) (Fig. 4b). Higher TMB entailed more mutated genes in MSI-H cancers (Extended Data Fig. 5b). Even with similar TMB, within the MSS group, i3 cancers were enriched for *KRAS* and *PIK3CA* mutations while i2 tumors were enriched in *APC* and *TP53* mutations (Fig. 4c).

Some of the expression differences between i2 and i3 epithelial cells may be directly attributable to differences in DNA copy number. Of the 715 DEGs, 382 coincided with the above-mentioned chromosomal arms commonly amplified or deleted in i2 tumors. For the majority (91%) of these 382 genes, expression fold-change direction was concordant with the difference in average copy number between i2 and i3, suggesting that their differential expression could be a direct consequence of DNA gain or loss (Fig. 4d). Genes upregulated in i2 tumors were enriched for MYC and E2F targets, perhaps reflecting i2-specific amplification of the chromosomal arms in which *MYC* (8q) and *E2F1* (20q) reside (Fig. 4a,e and Supplementary Fig. 7). Consistently, the MYC regulon defined by SCENIC showed higher expression in i2 epithelial cells (Fig. 2h). Genes upregulated in i3 cells were associated with Epithelial Mesenchymal Transition (EMT), inflammatory pathways and metabolic derangements (Fig. 4e, Extended Data Fig. 9, Supplementary Fig. 10 and Supplementary Tables 1–10).

### DNA methylation

Most MSI-H tumors showed a global CIMP. At the other extreme, none of the iCMS2_MSS tumors were hypermethylated. The CIMP status of iCMS3_MSS tumors was variable across patients, with a minority displaying DNA hypermethylation. Overall, we did not detect consistent epigenetic differences between iCMS2 and iCMS3 (Extended Data Fig. 5d).

### Cancer pathways

**Fig. 5:**
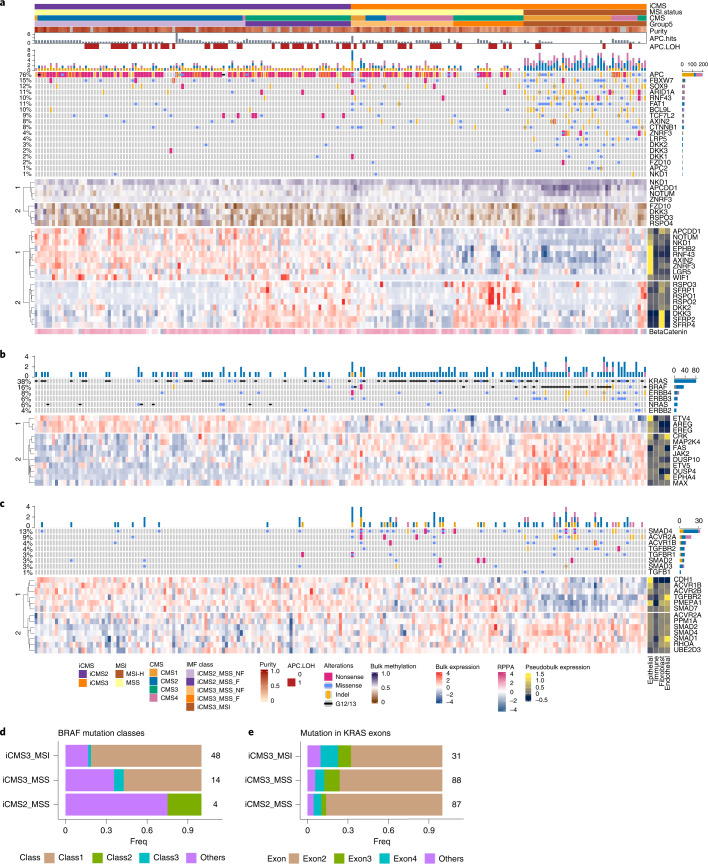
Relationship of iCMS and IMF to common cancer pathways. **a**–**c**, Heatmaps of mutation landscape (top), methylation (middle; **a** only) and bulk expression (bottom) of selected genes in the WNT (**a**), MAPK (**b**) and TGF-beta (**c**) pathways, across TCGA samples (*n* = 209). In the mutation Oncoprint, colors depict the type of mutation; a barplot of the cumulative frequency of each mutation is shown to the right, and the total frequency of mutations in each gene is shown to the left. The methylation heatmap is colored by beta-value, the gene expression heatmap is colored by scaled expression and the right annotation bar shows the average scaled expression of each gene across four major cell types (epithelial, immune, fibroblast, endothelial) from CRC-SG1 scRNA-seq data. In **a**, beta-catenin protein expression by reverse-phase protein arrays (RPPA) is displayed below the gene expression heatmap. **d**, Proportion of BRAF mutation classes in iCMS3_MSI (*n* = 48), iCMS3_MSS (*n* = 14) and iCMS2_MSS (*n* = 4) samples with BRAF mutations, in TCGA and SG-Bulk. **e**, Proportion of mutations in KRAS exons in iCMS3_MSI (*n* = 31), iCMS3_MSS (*n* = 88) and iCMS2_MSS (*n* = 87) samples with KRAS mutations, in TCGA and SG-Bulk. Number of samples in each group is labeled.

**Extended Data Fig. 6:**
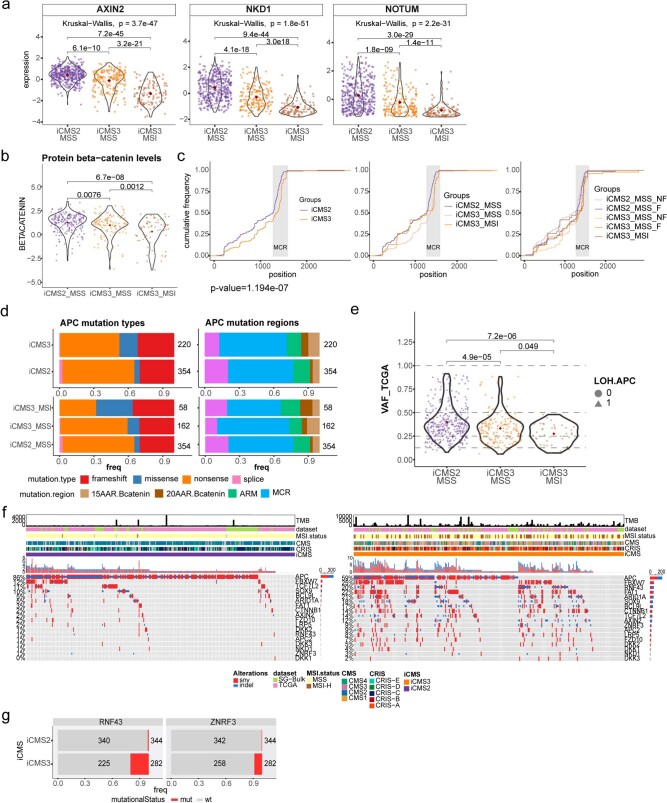
Wnt pathway alterations in iCMS. **a**. Scaled gene expression of selected Wnt pathway genes in iCMS2_MSS (n = 389), iCMS3_MSS (n = 195) and iCMS3_MSI (n = 116), from TCGA and SG-Bulk datasets. **b**. Beta-catenin protein levels, by RPPA, in iCMS2_MSS (n = 216), iCMS3_MSS (n = 117) and iCMS3_MSI (n = 67) from TCGA data. P-values for pairwise comparisons are by two-sided Wilcoxon rank-sum test with no correction; overall p-value is by Kruskal-Wallis test. **c**. Cumulative frequencies of truncating (nonsense and frameshift) APC mutations by position from TCGA and SG-Bulk patients with APC mutations (n = 342), in iCMS2 and iCMS3 (left), iCMS2-MSS vs iCMS3-MSS vs iCMS3-MSI (middle), as well as IMF 5 groups (right). P-value is by two-sided Kolmogorov–Smirnov test. **d**. Proportion of APC mutation types (left) and regions (right) in samples with APC mutations from TCGA and SG-Bulk data (n = 574). Comparisons are between iCMS2/iCMS3 (top), and iCMS2_MSS/iCMS3_MSS/iCMS3_MSI (bottom). **e**. APC variant allele frequency (VAF) in iCMS2_MSS (n = 343), iCMS3_MSS (n = 172) and iCMS3_MSI (n = 46) from TCGA data. P-values are by two-sided Wilcoxon rank-sum test with no correction **f**. Mutational landscape of selected Wnt pathway genes in iCMS2 (left, n = 344) and iCMS3 (right, n = 281) samples in TCGA and SG-Bulk datasets. **g**. Proportion of samples in TCGA and SG-Bulk datasets (n = 626) with wild type (wt) or mutations (mut) in RNF43 (top) and ZNRF32 (bottom); number of samples in each group is labelled.

We analyzed signaling pathways commonly dysregulated in CRC: WNT^16,17^, MAPK^18^ and TGF-beta^19^ (Fig. 5a–c and Supplementary Fig. 11). Genes within the WNT pathway tended to be upregulated in i2 bulk tumors, presumably due to their upregulation in i2 epithelial cells (Fig. 4e), which could in turn be attributable to increased activity of transcription factors mediating WNT signaling (*TCF7*, *ASCL2*)^20^ (Fig. 2h). Intriguingly, the i2-upregulated set included genes such as *NOTUM*, *AXIN2* and *NKD1* that suppress WNT signaling via negative feedback during normal tissue homeostasis^21^ (Fig. 5a and Extended Data Fig. 6a). Upregulation of these negative feedback regulators is presumably a consequence of WNT hyperactivity in i2 tumors^22,23^. Finally, protein beta-catenin abundance was significantly higher in i2 compared with i3 in the TCGA reverse-phase protein arrays data (Extended Data Fig. 6b).

We next examined somatic mutations in the WNT pathway (Extended Data Fig. 6c–g). While i3 tumors displayed diverse WNT mutations, i2 tumors were primarily characterized by inactivating *APC* mutations. In particular, *APC* mutations were significantly more proximal in i2_MSS than i3_MSS (*P* = 8 × 10^−07^; Extended Data Fig. 6c). More proximal *APC* mutations result in shorter, truncated proteins, associated with higher beta-catenin signaling^24,25^. The variant allele frequency of *APC* mutations was higher in i2_MSS than in i3_MSS (Extended Data Fig. 6e). In contrast, i3 tumors were enriched for other WNT mutations, including ligand-independent *CTNNB1* mutations, as well as ligand-dependent *RNF43* and *ZNRF3* mutations targeting the R-spondin 1 (RSPO1)-associated negative feedback loop^21^, especially in iCMS3_MSI (Extended Data Fig. 6f,g).

**Extended Data Fig. 7:**
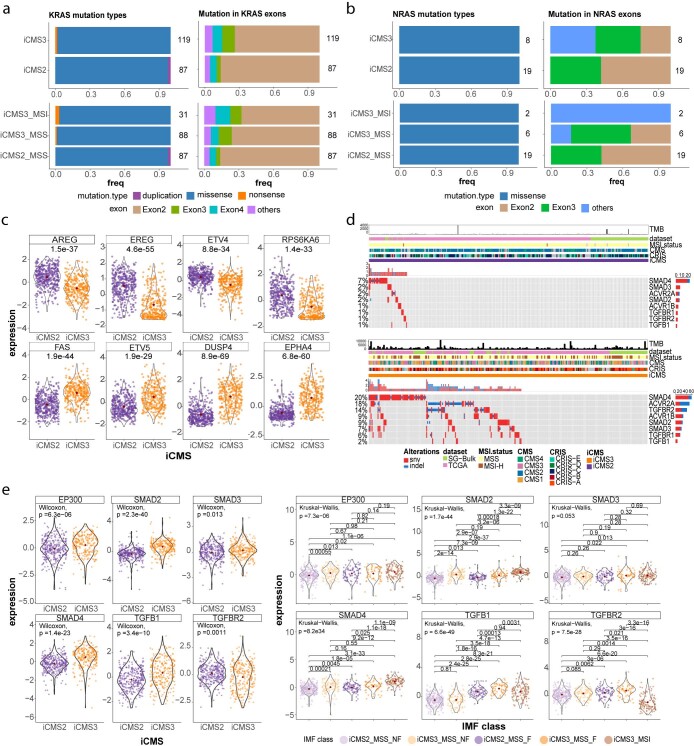
MAPK and TGF-beta pathways in iCMS. Proportion of mutation types and locations in **a**. KRAS (n = 206) and **b**. NRAS (n = 27) in samples with KRAS/NRAS mutations from TCGA and SG-Bulk data; number of samples in each group is labelled. Comparisons are between iCMS2/iCMS3 (top), and iCMS2_MSS/iCMS3_MSS/iCMS3_MSI (bottom). **c**. Scaled gene expression of selected MAPK pathway genes in iCMS2 (n = 396) and iCMS3 (n = 312), from TCGA and SG-Bulk datasets. P-values shown are by two-sided Wilcoxon rank-sum test without correction. **d**. Mutational landscape of selected TGF-b﻿eta pathway genes in iCMS2 (top, n = 344) and iCMS3 (bottom, n = 281) samples in TCGA and SG-Bulk datasets. **e**. Scaled gene expression of selected TGF-b﻿eta pathway genes iCMS2 (n = 396) and iCMS3 (n = 312) (left) and across the IMF 5 categories: i2_MSS (n = 240), i2_fibrotic (n = 82), i3_MSS (n = 92), i3_fibrotic (n = 58), i3_MSI (n = 105) (right). P-values are calculated using two-sided Wilcoxon rank-sum test without correction.

We next evaluated alterations associated with MAPK pathway upregulation in cancer. i3 cancers had more frequent *KRAS*, *PIK3CA* and *BRAF* mutations (Figs. 4c and 5b), including mutations known to be associated with more prominent MAPK pathway upregulation^26,27^. *BRAF* V600 class 1 mutations were only observed in i3 cancers and *KRAS* exon 3 mutations were enriched amongst i3 cancers (Fig. 5d,e and Extended Data Fig. 7a,b). i3 cancers showed higher expression of downstream MAPK components (*DUSP4* and *ETV5*) and i2 cancers had overexpression of *EGFR* ligands, *AREG* and *EREG* (Fig. 5b and Extended Data Fig. 7c). In i3 tumor cells, published gene signatures related to MAPK activity and *KRAS* and *BRAF* activating mutations^28–32^ were more highly expressed (Supplementary Fig. 12).

Upregulation of TGF-beta signaling was more prominent in i3 cancers. Genes in the TGF-beta signaling pathway, including *SMAD4*, were more frequently mutated in i3 cancers (Fig. 5c and Extended Data Fig. 7d). Expression of *SMAD2/3/4* is increased in i3 cancers (Extended Data Fig. 7e) but gene signatures related to TGF-beta activity^1,33–37^ were not consistently different in i3 and i2 tumor cells (Supplementary Fig. 11).

### Composition of tumor microenvironment

**Fig. 6:**
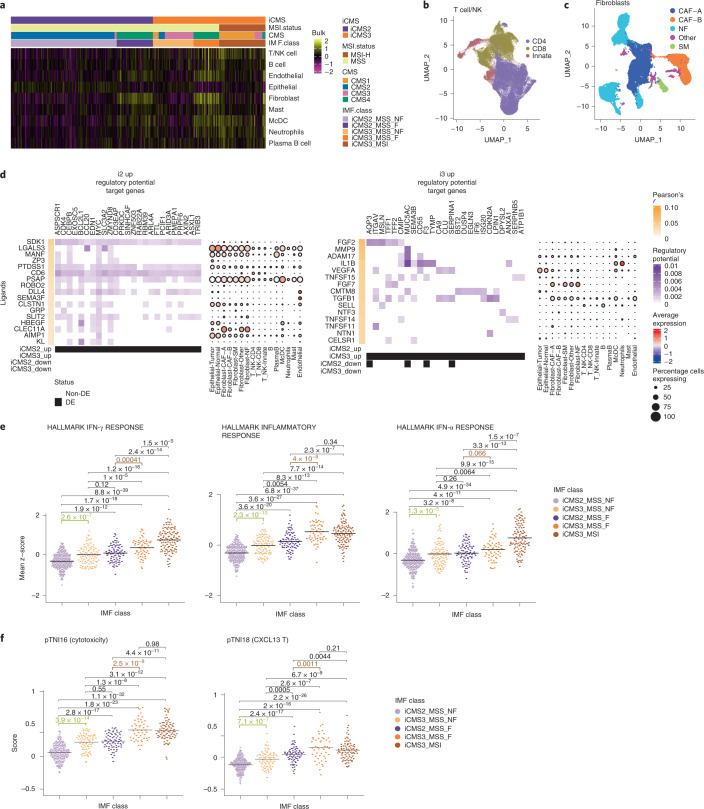
Epithelial cell interactions with microenvironment. **a**, Heatmap of the average scaled gene expression of cell-type-specific signatures of the nine major cell types in 577 bulk samples from TCGA and SG-Bulk datasets. **b**,**c**, UMAP of T cells (**b**) (*n* = 76,812 cells) and fibroblasts (**c**) (*n* = 31,451 cells) from 14 patients in CRC-SG1 dataset, colored by subtypes, used in signaling analyses. **d**, NicheNet analysis using i2 up gene set (left) and i3 up gene set (right). The heatmap depicts the regulatory potential scores (purple) for the top 200 target genes of each of the top 20 ligands ranked by Pearson correlation (orange) after filtering at a quantile cutoff of 0.33 for the regulatory potential score. The dotplot on the right depicts the average scaled patient-wise pseudo-bulk expression of each of the top-ranked ligands in each cell type across patients in the CRC-SG1 cohort. Dot size corresponds to the percentage of cells expressing the ligand in each cell type. **e**, Metascores for top three inflammation-related pathways identified by GSEA, in 577 bulk samples from TCGA and SG-Bulk, split by IMF: i2_MSS (*n* = 240), i2_fibrotic (*n* = 82), i3_MSS (*n* = 92), i3_fibrotic (*n* = 58), i3_MSI (*n* = 105). **f**, CXCL13 and cytotoxicity gene program scores (from Pelka et al. ^13^) in 462 bulk samples from TCGA, split by IMF: i2_MSS (*n* = 189), i2_fibrotic (*n* = 71), i3_MSS (*n* = 74), i3_fibrotic (*n* = 48), i3_MSI (*n* = 80). In **e** and **f**, *P* values are by two-sided Wilcoxon rank-sum test without adjustment of multiple comparison. DE, Differentially expressed; SM, Smooth Muscle.

**Extended Data Fig. 8:**
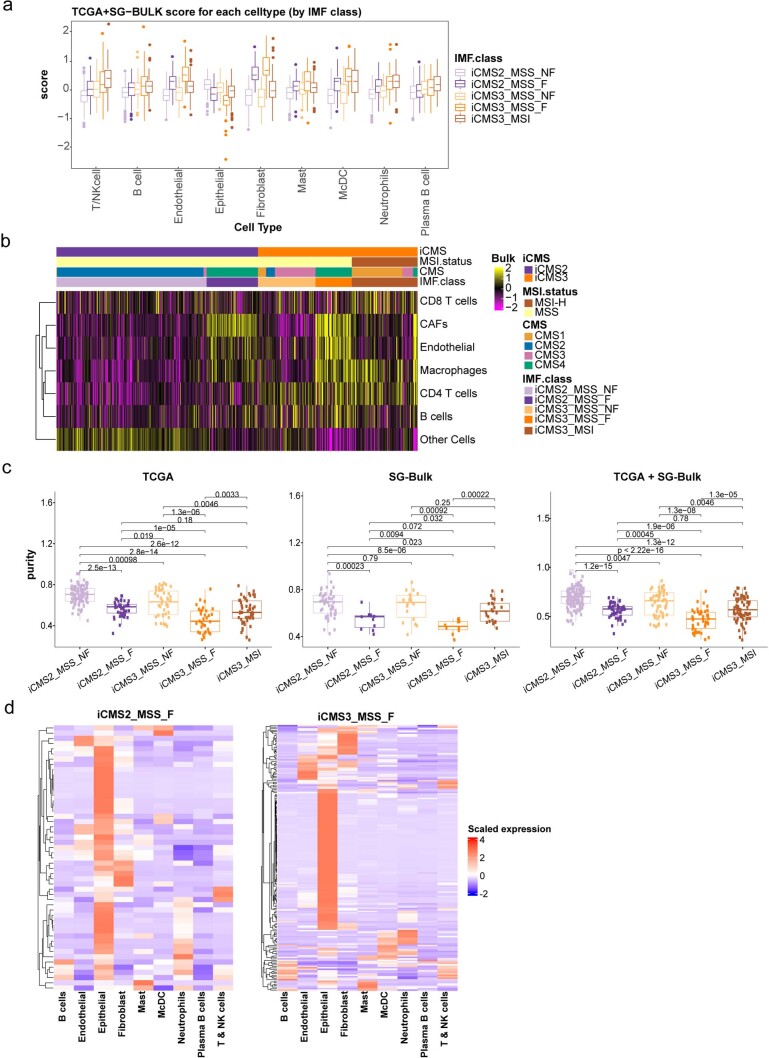
Tumor microenvironment in IMF classes. **a**. Boxplot of the average scaled gene expression of cell type-specific signatures of the 9 major cell types in 577 bulk samples from TCGA and SG-Bulk datasets, split by IMF. Center line indicate the median, and box edges indicate the 25th (Q1) and 75th (Q3) percentiles. Whiskers are plotted at 1.5xIQR and data beyond the end of the whisker are outliers. **b**. Heatmap of the EPIC cell fractions across the major cell types in TCGA and SG-Bulk datasets (n = 577). EPIC was performed on these datasets using the in-house cell type categories and reference panel^2^. EPIC scores were log-transformed, zero-centered and scaled to unit variance. Columns are patients ordered by IMF, and rows are cell types ordered by unsupervised hierarchical clustering. **c**. Tumor purity estimate of samples from TCGA (left), SG-Bulk (middle) and TCGA + SG-Bulk (right), split by IMF. TCGA: iCMS2_MSS (n = 96), iCMS2_fibrotic (n = 44), iCMS3_MSS (n = 42), iCMS3_fibrotic (n = 33), iCMS3_MSI (n = 49). SG-Bulk: iCMS2_MSS (n = 51), iCMS2_fibrotic (n = 11), iCMS3_MSS (n = 18), iCMS3_fibrotic (n = 10), iCMS3_MSI (n = 25). TCGA + SG-Bulk: iCMS2_MSS (n = 147), iCMS2_fibrotic (n = 55), iCMS3_MSS (n = 60), iCMS3_fibrotic (n = 43), iCMS3_MSI (n = 74). P-values are by two-sided Wilcoxon rank-sum test without correction. Center line indicate the median, and box edges indicate the 25th (Q1) and 75th (Q3) percentiles. Whiskers are plotted at 1.5xIQR and data beyond the end of the whisker are outliers. **d**. Mapping of differentially expressed genes between iCMS2_MSS_F and iCMS3_MSS_F onto CRC-SG1 pseudobulk expression matrix by cell type. The heatmap on the left shows genes upregulated in iCMS2_MSS_F compared to iCMS3_MSS_F, while the heatmap on the right shows genes upregulated in iCMS3_MSS_F compared to iCMS2_MSS_F.

**Extended Data Fig. 9:**
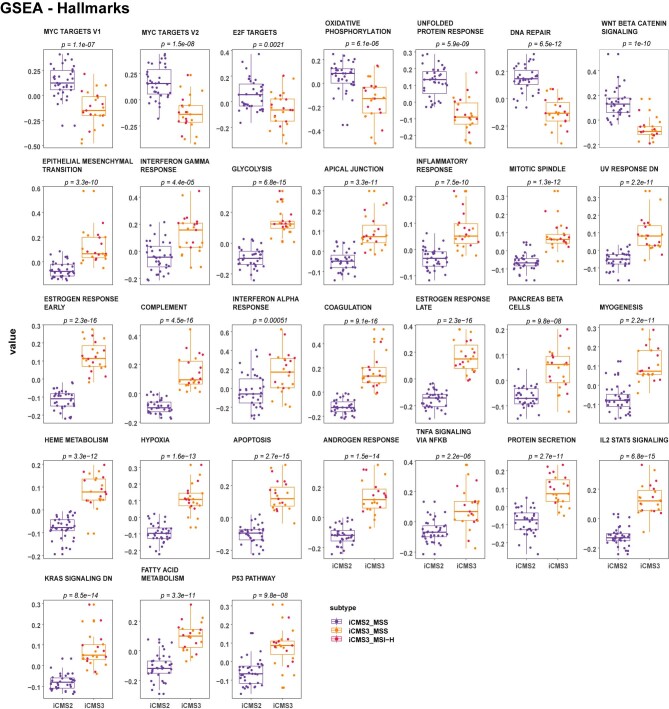
Comparison of metagene signature of selected GSEA-Hallmark pathway in single cell epithelial cells across iCMS classes. Box plots of metagene scores comparing iCMS2 (n = 35) versus iCMS3 (n = 23) in patient-specific pseudo-bulk. Within the iCMS3 group, i3-MSI (n = 10) samples are labelled by red jitter points and i3-MSS samples are labelled by orange jitter points. The metagene scores for each patient pseudo-bulk was calculated by averaging the scaled expressions of all genes in the geneset in the given patient. P-values were calculated by two-sided Wilcoxon rank-sum test. Center line indicate the median, and box edges indicate the 25th (Q1) and 75th (Q3) percentiles. Whiskers are plotted at 1.5xIQR and data beyond the end of the whisker are outliers.

To compare cell-type abundance across tumor types, we identified marker genes for nine major fibroblast, immune and endothelial cell types (Extended Data Fig. 2b) from CRC-SG1 single-cell data, and calculated the metagene expression score for each cell type by averaging its markers in bulk transcriptomes (Supplementary Notes). Consistent with previous reports, we observed higher T/natural killer (NK) cell scores in MSI-H tumors, and elevated fibroblast, endothelial and monocyte/classical dendritic cell (McDC) scores in fibrotic (CMS4) tumors (Fig. 6a and Extended Data Fig. 8a)^38^. However, within fibrotic tumors, we observed higher T/NK, McDC and neutrophil metagene scores in iCMS3_MSS_F than in iCMS2_MSS_F (Fig. 6a and Extended Data Fig. 8a). EPIC cell-type deconvolution^39^ produced similar results (Extended Data Fig. 8b). We used matched exome sequencing data to estimate sample tumor purity. While fibrotic tumors had lower tumor purity, iCMS3_MSS_F still had decreased tumor purity compared with iCMS2_MSS_F (Extended Data Fig. 8c).

Next, we performed bulk tumor DEG analysis of iCMS3_MSS_F compared with iCMS2_MSS_F. Genes upregulated in iCMS3_MSS_F were specific to McDC and endothelial cells in our single-cell data (Extended Data Fig. 8d). Thus, in the fibrotic context, iCMS3 tumors are associated with increased fibroblast, myeloid and endothelial cell signatures and decreased tumor purity, suggesting that, when fibrosis develops, iCMS3 tumors have a larger fibrotic reaction than iCMS2 tumors. In addition, in the nonfibrotic context, iCMS3 tumors show increased immune cell signatures (T/NK, plasma-B cell, McDC and granulocyte) compared with iCMS2 (Fig. 6a and Extended Data Fig. 8a).

### Cell signaling interactions

We examined tumor epithelial cell signaling with immune cells, endothelial cells and fibroblasts^8^ (Extended Data Fig. 2 and Fig. 6c) using NATMI^40^ and Nichenet^41^ (Fig. 6d and Supplementary Fig. 8a). Signaling pathway target genes with the highest regulatory potential scores and top ligands prioritized by NicheNet included key regulons previously identified in our regulon analysis, such as *CEBPB*, *ARID3A* and *MYC* in i2. For i3, top ligands included *FGF2* and *IL1B*, reported to promote invasiveness in CRC^42,43^. Using NATMI, we ranked signaling interactions and inspected top ligand–receptor combinations (Supplementary Fig. 8b and Supplementary Table 11). Differential interactions stronger in i2 included EREG-to-EGFR signaling from epithelial tumor cells to epithelial tumor cells (autocrine) and fibroblasts (paracrine) (Supplementary Fig. 8c). Multiple immune–epithelial interactions involving T/NK cells and McDCs with tumor epithelial cells were predicted in i3 cancers (for example, *IL1B* in McDCs to *IL1R2* in i3 tumor cells) (Supplementary Fig. 8c).

### Immune response

Our signaling analyses suggested pro-inflammatory interactions in i3 tumors. The NFKB1 regulon, associated with inflammation, was upregulated in i3 (Fig. 2h). We observed an increase in immune cell signatures, including T/NK cells, McDCs and neutrophils, in iCMS3_MSS tumors, in both the fibrotic and nonfibrotic settings (Fig. 6a).

Epithelial gene set enrichment analysis (GSEA) identified multiple immune pathways amongst the top pathways upregulated in i3 cells, including ‘INTERFERON GAMMA RESPONSE’, ‘INFLAMMATORY RESPONSE’ and ‘INTERFERON ALPHA RESPONSE’. We calculated metagene scores using the GSEA leading edge genes for these three top inflammation-related pathways in our bulk dataset (Fig. 6e). MSI-H and fibrotic (CMS4) tumors had higher expression of inflammation-related pathways^38^. Notably, in both the fibrotic and nonfibrotic settings, i3 tumors had higher expression of inflammation-related pathways than i2 tumors.

A recent single-cell CRC study^13^ identified T cell program activity scores that were different between MSI-H and MSS CRCs. We hypothesized that iCMS would provide a further substratification of increased immune activity within MSS tumors. In TCGA bulk transcriptomes, we quantified the expression of two T cell programs of anti-tumor reactivity and effector function (‘CXCL13 T cell’ and ‘Cytotoxicity’ programs) that this study had identified as differentially active between MSI-H and MSS CRCs (Fig. 6f). In both fibrotic and nonfibrotic contexts, iCMS3_MSS tumors were associated with a more inflammatory, immune-activated environment than iCMS2_MSS tumors, with levels in the iCMS3_MSS_F group similar to iCMS3_MSI tumors. Our single-cell and bulk analyses point to iCMS3_MSS as a unique subset of MSS CRCs, with similarities to MSI-H tumors, increased immune activation, and higher signatures of T and myeloid cell infiltration and anti-tumor cytotoxicity.

### Pre-invasive and cell lineage gene sets

**Fig. 7:**
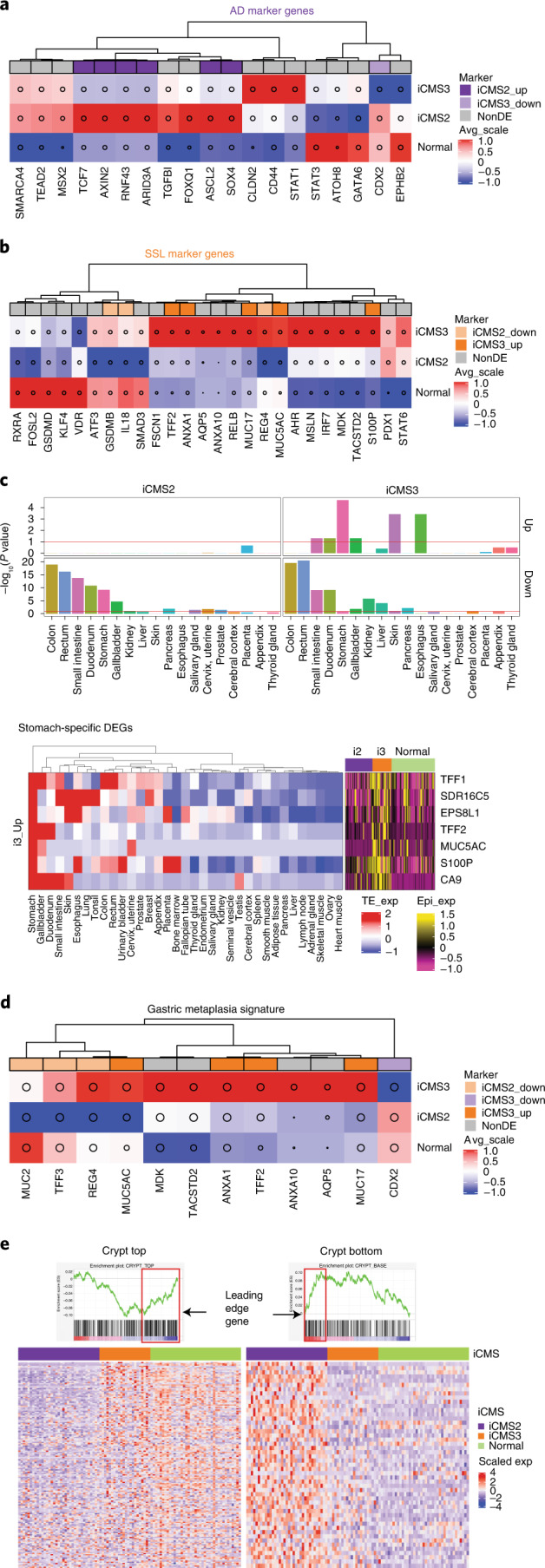
Association of iCMS markers with polyp subtypes and normal tissues. **a**,**b**, Heatmaps of tubular adenoma (AD) (**a**) and SSL (**b**) marker genes obtained from Chen et al. ^44^, colored by the average of scaled (z-transformed) expression values of epithelial single cells from five-cohort scRNA-seq data (patients = 61). **c**, Barplots quantify enrichment of tissue-specific genes in each of the four DEG sets, calculated using the TissueEnrich package (iCMS2 Up: 308; iCMS2 Down: 279; iCMS3 Up: 74; iCMS3 down: 54; total: 715). Red line, *P* = 0.1. The heatmaps show expression levels of the seven iCMS3-Up DEGs defined as stomach-specific in the TissueEnrich database. Left, scaled expression in diverse tissues. Right, scaled epithelial pseudo-bulk expression in 61 patients. **d**, Heatmap of gastric metaplasia signature genes, similar to **a** and **b. e**, Heatmap of GSEA leading edge genes within crypt top and crypt bottom gene sets, showing scaled epithelial pseudo-bulk expression levels across 61 patients from five scRNA-seq cohorts.

**Extended Data Fig. 10:**
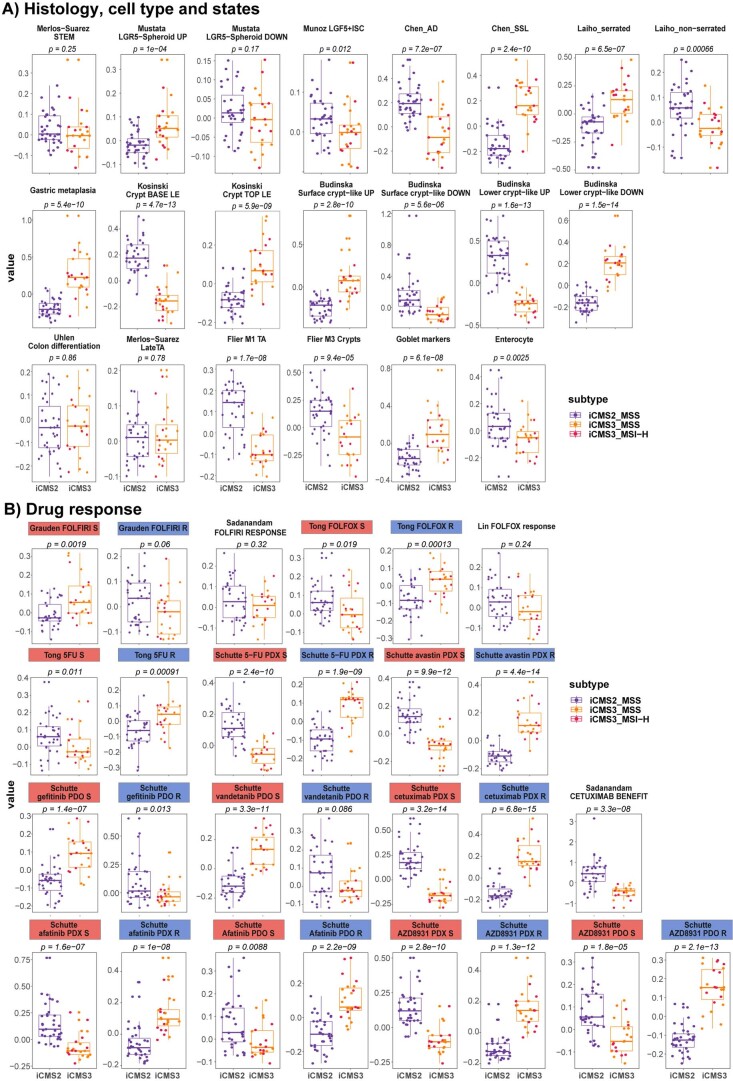
Comparison of metagene signature of selected histology, cell types & states of the colon (A) and drug response signatures (B) in single cell epithelial cells across iCMS classes. Box plots of metagene scores comparing iCMS2 (n = 35) versus iCMS3 (n = 23) in patient-specific pseudo-bulk. Within the iCMS3 group, i3-MSI (n = 10) samples are labelled by red jitter points and i3-MSS samples are labelled by orange jitter points. The metagene scores for each patient pseudo-bulk was calculated by averaging the scaled expressions of all genes in the geneset in the given patient. P-values were calculated by two-sided Wilcoxon rank-sum test. Center line indicate the median, and box edges indicate the 25th (Q1) and 75th (Q3) percentiles. Whiskers are plotted at 1.5xIQR and data beyond the end of the whisker are outliers.

Recently, a pre-cancer atlas study identified two cell types^44^, one attributable to adenomatous polyps and one to sessile serrated lesions (SSLs). In our single-cell data, most adenomatous polyp markers showed higher expression in iCMS2 patients, whereas SSL markers were upregulated in iCMS3 (Fig. 7a,b and Extended Data Fig. 10a). The previous study noted that SSLs were highly enriched for genes not normally expressed in the colon, and suggested that gastric metaplasia may underlie their etiology^44^. We therefore hypothesized that iCMS3 tumors may also show the same trend. Indeed, TissueEnrich^45^ analysis indicated that the genes upregulated in iCMS3 epithelial cells (iCMS3 Up) were significantly enriched for stomach-specific expression, and also for expression in other foregut tissues such as esophagus and duodenum (Fig. 7c). Similarly, genes related to gastric metaplasia were upregulated in iCMS3 epithelial cells (Fig. 7d and Extended Data Fig. 10a), suggesting that this process might be active within i3 cancers. Genes specific to normal colon were preferentially downregulated in both i2 and i3 tumors, suggesting loss of differentiation in oncogenesis (Fig. 7c and Extended Data Fig. 10a). Furthermore, inspecting the expression of the leading edge genes in our GSEA, we also observed upregulation of crypt top genes in i3 cancers and crypt bottom genes in i2 cancers (Fig. 7e and Extended Data Fig. 10a). These results suggest that i3 tumors show gastric metaplasia and may arise from malignant transformation of SSLs, which in turn could originate from cells resembling crypt top. Conversely, i2 tumors may arise from cells resembling crypt bottom, progressing via adenomatous polyps before becoming full-blown cancers.

### Drug response of iCMS classes

We classified iCMS status of cell lines from the CTRPv2 dataset^46^ and observed that i2 and i3 cell lines showed differential sensitivity to numerous drugs (Supplementary Fig. 9a,b). However, the difference in sensitivity to standard-of-care chemotherapeutics such as Fluorouracil (5-FU), oxaliplatin and 7-ethyl-10-hydroxycamptothecin (SN-38) was not significant. We then evaluated two sets of genes whose expression was correlated with drug response (drug response signatures)^47–50^. For Folinic acid, fluorouracil and oxaliplatin (FOLFOX), 5-FU, avastin, cetuximab, afatinib and AZD8931, gene sets positively correlated with drug sensitivity were upregulated in iCMS2 cells and genes correlated with drug resistance were downregulated. Similarly, iCMS3 cells showed patterns of up- and downregulation suggesting responsiveness to FOLFIRI, gefitinib and vandefanib (Extended Data Fig. 10b). This prompts investigation of potentially differential drug responses based on iCMS status in retrospective analyses of tumor samples from completed clinical trials.

## Discussion

**Fig. 8:**
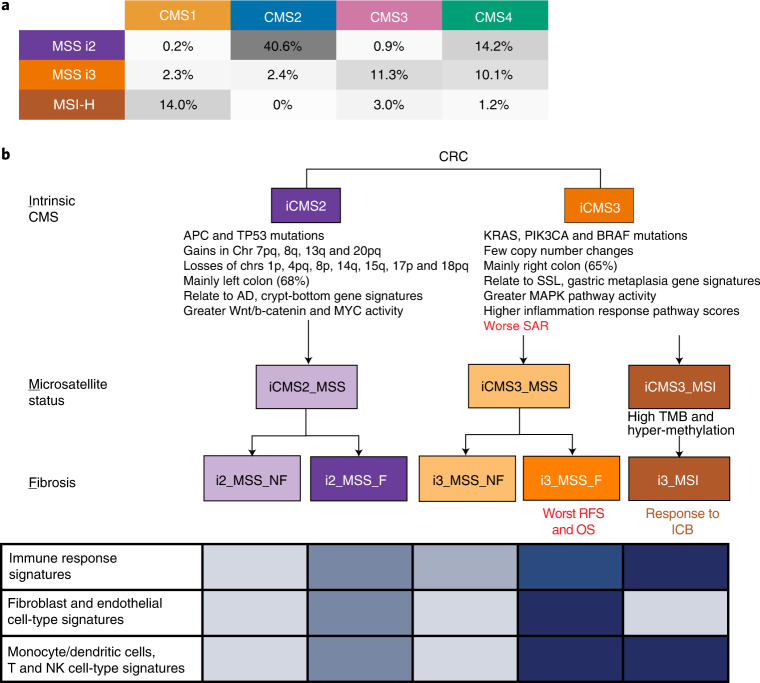
The proposed IMF classification of CRC. **a**, Percentage of samples (*n* = 577) from TCGA/SG-Bulk/CMS cohorts with complete CMS/iCMS/MSI calls broken down by epithelial traits as rows (intrinsic epithelial subtype, microsatellite instability status) and bulk tumor CMS classification as columns. The five most frequent combinations, which account for 520 of 577 samples (~90%), are indicated as shaded and defined as IMF subtypes in **b.** **b**, Schematic model of IMF classification comprising a sequential layered classification based on intrinsic CMS subtypes, MSI status and fibrosis (as represented by CMS4), key clinico-molecular features, immune response signatures and single-cell-derived cell-type-specific signatures. Color intensity in table: expression rank in Fig. 6e,f (immune response), Fig. 6a and Extended Data Fig. 8 (fibroblast, endothelial, McDc, T and NK), with the darkest color denoting strongest expression. ICB, immune checkpoint blockade.

The CMS subtypes were discovered using bulk transcriptomics, leaving the underlying cellular phenotypes unresolved. We identified a central epithelial backbone of CRC, iCMS2 and iCMS3, each characterized by distinct copy number genetics, transcriptional profiles and gene regulatory units. Based on this, we refined the CMS classification with five functional units of CRC through the IMF classification, a sequential layered structure comprising intrinsic epithelial subtype, microsatellite status and presence of fibrosis (Fig. 8). We have summarized the key biological and clinical characteristics of the five IMF tumor classes in Supplementary Table 12.

Only MSI-H CRCs respond to anti-PD1 immunotherapy^13,51^. Immunotherapy response is influenced by neoantigen quality and quantity^52^, epithelial cell intrinsic properties^53^ and immunological nodes^54^ within the tumor–immune microenvironment. MSI-H cancers have a high TMB, including frameshift mutations. Despite a lower TMB, iCMS3_MSS tumors are more similar transcriptomically and in gene regulatory networks to MSI-H colon cancers than to iCMS2_MSS. Characterizing their distinct immunology could enable prioritization of customized immunotherapy combinations for clinical development^55^ in i2_MSS and i3_MSS cancers.

Fibrotic CMS4 CRCs, previously assumed to be a coherent group, actually comprise two epithelial subtypes. Amongst fibrotic tumors, iCMS3_MSS_F has the greatest propensity to metastasize. Fibroblast, myeloid and endothelial cells appear to be enriched in such tumors. Mechanistic analysis of iCMS3_MSS_F could enable biologically directed therapies to prevent metastases in this subgroup with the worst RFS. Our results also indicate that intrinsic epithelial subtype and fibrosis are decoupled^56^, and prompt future studies to characterize drivers of the ‘switch’ to fibrosis in i2 and i3 epithelial contexts.

We have focused on neoplasms that ‘successfully’ progressed to established cancers. The dichotomous biology of i2 and i3 cancers might have resulted from distinct developmental origins and trajectories^57^. Gene set analysis suggests distinct preneoplastic polyps of origin, with i2 cancers potentially arising from adenomatous polyps and i3 from serrated polyps^44^. This connects intrinsic subtypes to the classical and serrated pathways of tumorigenesis^58^, with some biological features retained in established tumors. i2 cancers displayed a crypt bottom signature while i3 cancers exhibited crypt top and gastric metaplasia signatures. i2 cancers likely developed through initial expansion from an LGR5^+^ crypt bottom stem cell, acquiring copy number alterations and aneuploidy accelerated by *TP53* mutations and characterized by prominent WNT pathway activation from an early loss-of-function *APC* mutation. The origin of i3 cancers is less clear. A committed crypt top progenitor cell, perhaps due to repeated mucosal injury^57^, may have undergone dedifferentiation, with disordered gastric metaplasia, accompanied by increasing inflammation contributing to the development of an i3 cancer. Experimental studies could elucidate the oncogenic trajectories of i2 and i3 tumors, and would have implications for cancer prevention, screening and detection.

## Methods

### Patient and tissue sample collection

The study was approved by the institutional review boards of Singhealth (2018-2795 and 2018-2376) for CRC-SG1 and CRC-SG2, Samsung Medical Center (approval no. SMC2017-07-131) for the SMC and Commissie Medische Ethiek UZ KU Leuven/Onderzoek (approval no. S50887-ML4707) for the KUL3 and KUL5 datasets, respectively. All mentioned datasets/studies were carried out in accordance with ethical guidelines and all patients provided written, informed consent. The study involves 26 Singaporean, 23 Korean and 14 Belgian patients diagnosed with CRC who underwent surgery (Supplementary Table 13). For CRC-SG1, KUL3 and KUL5 cohorts, additional samples per patient were obtained from multiple sites. After resection, samples from both tumor and nonmalignant colon tissues were collected and immediately transferred for tissue preparation. Tissues were subjected to single-cell isolation, AllPrep DNA/RNA Mini Kit (QIAGEN) for DNA analysis and transcriptome sequencing.

### scRNA-seq sample preparation

For both CRC-SG1 and CRC-SG2 samples, tissue specimens almost 10 mm^3^ in dimensions were processed similarly to the KUL samples. The transportation medium (RPMI with 10% FBS) was decanted out and the tissue specimens were weighed and placed on ice in petri dishes. Tissues were then subjected to fine mincing in 5 ml of RPMI solution (with 10% FBS) using sharp and sterile scalpel blades to make a fine slurry. The minced tissue was resuspended in pre-warmed Dissociation buffer 2X, comprising Collagenase-P (4 mg ml^−1^, Roche) and DNase-I (0.4 mg ml^−1^, Roche) in a total of 5 ml of RPMI medium containing 10% FBS (Gibco, Life Technologies). The tissue suspension was subjected to two rounds of shaking incubation of 6 min each with 1 min of vigorous vortexing in between. The enzymatic digestion step was followed by one more round of physical homogenization by pipetting the tissue suspension through the 10-ml and 5-ml pipette bores for at least 1 min. The resulting suspension was then washed with ice-cold Buffer I (BSA 1%/DPBS/EDTA (2 mM)) and the contents were passed through a 70-µm strainer to get rid of the un-dissociated mass. The filtrate (single-cell suspension) was centrifuged at 500*g* for 5 min at 4 °C. The supernatant was decanted and the cell pellet subjected to red blood cell lysis using the ACK lysis buffer (Gibco, Life Technologies) for 5 min on ice. The cell pellet was again washed with Buffer I and re-filtered through a 40-µm strainer and centrifuged at 300*g* for 5 min at 4 °C. Later, the supernatant was decanted without disturbing the pellet and resuspended in 3–5 ml of RPMI depending upon pellet size. Cells were subsequently assessed for viability and concentration using trypan blue on a disposable cell calculator (C-Chip, Countess chip, Digital Bio).

For KUL5, samples were rinsed with PBS, minced on ice to pieces of <1 mm^3^ and transferred to 10 ml of digestion medium containing Collagenase-P (2 mg ml^−1^, ThermoFisher Scientific) and DNAse-I (10 U µl^−1^, Sigma) in DMEM (ThermoFisher Scientific). Samples were incubated for 15 min at 37 °C, with manual shaking every 5 min. Samples were then vortexed for 10 s and pipetted up and down for 1 min using pipettes of descending sizes (25, 10 and 5 ml). Next, 10 ml of ice-cold PBS containing 2% FBS was added and samples were filtered using a 40-µm nylon mesh (ThermoFisher Scientific). Following centrifugation at 500*g* and 4 °C for 5 min, the supernatant was decanted and discarded, and the cell pellet was resuspended in red blood cell lysis buffer. Following a 5-min incubation at room temperature, samples were centrifuged (500*g*, 4 °C, 5 min) and resuspended in 1 ml of PBS containing 8 µl of UltraPure BSA (50 mg ml^−1^; AM2616, ThermoFisher Scientific) and filtered over Flowmi 40-µm cell strainers (VWR) using wide-bore 1-ml low-retention filter tips (Mettler-Toledo). Next, 10 µl of this cell suspension was counted using an automated cell counter (Luna) to determine the concentration of live cells.

### scRNA-seq library preparation and data processing

For CRC-SG1, CRC-SG2, KUL3 and KUL5 datasets, fresh single-cell suspensions were loaded into the Chromium system (10X Genomics) targeting 5,000 cells per well. For the SMC dataset, the cryopreserved single-cell dissociate was rapidly thawed, washed and loaded in the same fashion. Barcoded sequencing libraries were generated using the Chromium Single Cell 3′ v2 Reagent Kit (SMC, KUL3), 3′ v3 Reagent Kit (CRC-SG2) or 5′ Reagent Kit (CRC-SG1, KUL5). All libraries were sequenced on an Illumina NextSeq 500, HiSeq 4000 or NovoSeq 6000 until sufficient saturation was reached. After QC, raw sequencing reads were aligned to the human reference genome, GRCh38, and processed using CellRanger v.3.1.

### MSI status determination for CRC-SG1, CRC-SG2, KUL3, KUL5 and SMC

For the SG-CRC1 and SG-CRC2 datasets, MSI status was determined by immunohistochemistry for MLH1, MSH2, MSH6 and PMS2. For KUL3, SMC and KUL5 datasets, MSI status was determined using the MSI Analysis System v.1.2 (Promega Corporation).

### SG-Bulk cohort: DNA/RNA extraction and sequencing, mutational and transcriptome analyses

We performed DNA and RNA sequencing on 151 patients with CRC from Singapore (SG-Bulk). The study was approved by the institutional review board of Singhealth (2018-2795). Ten 5-µm tissue sections were cut using standard microtomy techniques. Each collected cell population was then extracted using the Allprep kit. Extracted material was then quantified using a Qubit fluorometer. DNA-sequencing libraries were captured to exome regions using xGen Exome Research Panel v.1.0 (IDT), and libraries were prepared using the KAPA HyperPrep Kit. DNA libraries were sequenced to a target depth of ×200 for tumor samples and ×100 for normal samples on the Illumina HiSeq platform. RNA-sequencing libraries were prepared using the KAPA Stranded RNA-Seq Kit with RiboErase (Kapa Biosystems) and sequenced to a target depth of 200 million reads on the Illumina HiSeq platform (Illumina). RNA samples were aligned to the RefSeq build 73 transcriptome using Bowtie2 v.2.2.6 and quantified using RSEM v.1.2.2528. Gene expression was quantified using Salmon v.0.9.1 with hg19 reference from Ensembl v.75.

### Mutation

Sequencing data were processed by the bcbio-nextgen pipeline. Briefly, sequencing reads were aligned to the human genome (hg19) using the Burrows–Wheeler Aligner (v.0.7.17) and preprocessed using the Genome Analysis Toolkit 4 (GATK4, v.4.0.2.1). Somatic mutations were first called by four independent mutation callers: VarScan^59^, MuTect^60^, VarDict^61^ and FreeBayes^62^, using default parameters of the bcbio-nextgen pipeline. The final list of high-confidence mutations were called using a random forest-based ensemble mutation caller, SMuRF^63^, from the output of the four mutation callers. Copy numbers were estimated by CNVKit^64^ using default parameters of the bcbio-nextgen pipeline. Tumor purities were estimated using PurBayes^65^, ASCAT^66^, ESTIMATE^67^ and AbsCN-seq^68^, and consensus tumor purities were calculated using the mean of the tumor purity estimates^69^ available. Purity-adjusted copy numbers were calculated with the consensus tumor purity estimates.

TCGA and TCGA CNV data were obtained from: https://gdc.cancer.gov/about-data/publications/pancanatlas.

Loss of heterozygosity data for TCGA^70^ were obtained from the ‘ABSOLUTE-annotated seg file’: https://api.gdc.cancer.gov/data/0f4f5701-7b61-41ae-bda9-2805d1ca9781.

APC variant allele frequency was determined as VAF = t_alt_count/(t_ref_count + t_alt_count); t_ref_count: read depth supporting the reference allele in tumor; t_alt_count: read depth supporting the variant allele in tumor.

TCGA RNA-sequencing data were obtained from TCGA-COAD and TCGA-READ, workflow HTSeq-FPKM from https://portal.gdc.cancer.gov/repository. Thirteen Robust Multichip Average/Frozen robust multiarray analysis (RMA/FRMA) normalized microarray datasets^1^ were obtained from https://www.synapse.org/#!Synapse:syn2634742.

TCGA Illumina 450K methylation data were obtained from TCGA-COAD and TCGA-READ, data category ‘dna methylation’, platform ‘illumina human methylation 450’ from https://portal.gdc.cancer.gov/repository.

### scRNA-seq data: QC and defining major cell types

Raw scRNA-seq reads were assigned to cells (barcoded droplets) using CellRanger v.3.1 to generate the raw expression matrix of unique molecular identifier counts (UMI counts; indicative of number of unique RNA molecules detected) for each gene in each cell (droplet). Droplets with number of detected genes (NODG) < 300 were discarded as empty droplets. UMI counts were then normalized so that each cell had a total of 10,000 UMIs across all genes and these normalized counts were log-transformed with a pseudocount of 1 using the LogNormalize function in the Seurat package. Each log-transformed single-cell transcriptome was then projected using Pearson correlation onto the reference transcriptomes in the Global Panel of the RCA2 supervised clustering algorithm^8^. Cells were then clustered by RCA2 in this projection space using Seurat graph-based clustering. To identify cell clusters, we used ‘estimateCellTypeFromProjectionPerCluster’ from RCA2 with default parameter settings. This procedure was performed individually on the cells from each cohort to identify the following major cell types: B, endothelial, entericglial, epithelial, fibroblast, mast, McDC, neutrophil, Plasmacytoid dendritic cells (pDC), plasma-B and T/NK cells.

In a second round of QC, we calculated the median NODG across cells. For each major cell type, we then calculated the median of these medians across all samples and defined this as the reference NODG for that cell type. If a sample’s cell-type-specific median NODG deviated by more than a factor of 2 from the reference NODG for that cell type, this was counted as a substantial deviation in data quality. If a sample showed substantial deviations for more than half of the cell types (that is, six or more cell types), it was defined as a low-quality sample and discarded from the dataset. In the end, 22 samples from nine patients were discarded (Extended Data Fig. 1).

After that, we removed all doublets in each sample by running DoubletFinder v.2.0.3 (ref. ^71^). In a nutshell, DoubletFinder can be broken up into four steps: generate artificial doublets, pre-process merged real–artificial data, perform PCA and use the principal component distance matrix to find each cell’s proportion of artificial *k*-nearest neighbors (pANN), and finally rank order and threshold pANN values according to the expected number of doublets. Here, we followed the suggested workflow written in the author’s tutorial pages, with doublet rates of 0.8% per 1,000 recovered cells (following 10X Genomics protocol).

In the last round of QC, we finally applied a stringent set of major cell-type-specific QC cutoffs to NODG and the percentage of mitochondrial reads^72^ to define the final set of 49,155 epithelial cells from five cohorts (Extended Data Fig. 1b).

### Unsupervised (de novo) subclustering of epithelial cells in CRC-SG1 cohort

To cluster the 15,920 high-quality epithelial cells described above, we first used DUBStepR, a correlation-based feature selection algorithm that outperforms existing methods across diverse clustering benchmarks, to identify an informative set of genes^10^ (Supplementary Fig. 1). Here, we used num.pcs = 15 and min.cells = 160 (1% of all cells), while other parameters were left at default values. Since samples from 3 of the 14 patients (CRC2783, CRC2786 and CRC2787) were processed in a separate batch, we ran DUBStepR twice, once on datasets from the latter three and once on the remaining datasets, and then used the union of the two sets of feature genes for downstream de novo graph-based Louvain clustering using Seurat^73^ at resolution 0.2. To further refine cluster assignment, we identified the set of DEGs between all possible pairs of cell clusters (pairwise-DEGs). To identify pairwise-DEGs, we ran a modified version of the Findmarkers function in Seurat by changing the multiple testing correction method to Benjamini–Hochberg and applying a minimum average expression threshold of 0.1 for the upregulated group. We also set logfc.threshold = 0.4055 (corresponding to a fold-change of 1.5), min.pct = 0.25 and p_val_adj < 0.05. We then defined pairwise-DEGs as the union of the 30 most significant upregulated DEGs and the 30 most significant downregulated genes for each pairwise comparison. Using this new feature set, we re-clustered the cells using Seurat with resolution 0.5 (Fig. 2a and Extended Data Fig. 2a).

### Inter-patient transcriptomic heterogeneity in epithelial cells from CRC-SG1 cohort

Based on the above refined set of epithelial cell subclusters in the CRC-SG1 cohort, we used pairwise-DEG analysis once again to identify 823 DEGs. We then discarded the six clusters representing normal-like epithelial cells: clusters 2, 4, 7, 8, 16 and 17 (Extended Data Fig. 2a) and averaged the transcriptomes of all remaining tumor-like epithelial cells within each patient (all sectors) to define 14 patient-specific ‘pseudo-bulk’ tumor transcriptomes. We visualized these 14 pseudo-bulk transcriptomes by performing PCA on the 823 pairwise-DEGs and plotting the first two principal components. As in all PCA and clustering analyses in this study, we zero-centered and scaled each gene to unit variance before PCA. This revealed two major epithelial cell subtypes, which we defined as intrinsic CMS subtypes 2 and 3 (i2 and i3; Fig. 2b).

We then constructed up to three sample origin-specific pseudo-bulk transcriptomes for each patient: normal, primary tumor, lymph node. These were then grouped by epithelial subtype into i2 (primary, lymph), i3 (primary, lymph) and normal-like (primary, lymph, normal) pseudo-bulk transcriptomes, followed by pairwise-DEG analysis as before between the three subtypes: i2, i3, normal-like. Markers for each subtype were defined as genes that were significantly upregulated relative to both of the other subtypes. This resulted in 848 epithelial subtype-specific markers in total for CRC-SG1 i2, i3 and normal-like cells (CRC-SG1_iCMS2_Up: 368 genes; CRC-SG1_iCMS3_Up: 141 genes; CRC-SG1_normal_Up: 339 genes; Supplementary Fig. 2a).

### Clustering and visualization of epithelial cell transcriptomes from four additional cohorts

We used the 848 epithelial subtype-specific markers from CRC-SG1 to cluster each cohort individually using Seurat (Supplementary Fig. 2b). We also visualized epithelial transcriptomes from each of the four cohorts using PCA of patient-specific epithelial pseudo-bulk transcriptomes (Supplementary Fig. 2c). To reduce cohort-specific batch effects in joint analysis of single-cell data from the five cohorts, we zero-centered and scaled each gene to unit variance (epithelial cells only) within each cohort before combining epithelial cells across the five cohorts. We then used Louvain graph-based clustering as implemented in Seurat to identify epithelial cell subtypes in the merged dataset (Fig. 2c).

### Association between iCMS classification of epithelial cells and CMS subtypes based on bulk transcriptomes

We obtained 693 CMS marker genes from the previously developed^1^ CMSclassifier tool (https://github.com/Sage-Bionetworks/CMSclassifier), of which 666 were expressed (nonzero in at least one cell) in our five-cohort epithelial scRNA-seq dataset. Each of the 666 genes was defined as a marker of the CMS subtype in which it showed highest expression (CMSclassifier: centroids.RData). In this manner, we obtained 215, 122, 135 and 194 marker genes for CMS1, CMS2, CMS3 and CMS4, respectively (Supplementary Fig. 3). We then analyzed tumor scRNA-seq data to assign these bulk CMS markers to specific cell types. For each patient, we first averaged across single cells from tumor samples to define the patient-specific pseudo-bulk transcriptomes of B cells, endothelial, entericglial, epithelial, fibroblast, mast, McDC, neutrophils, Plasmacytoid dendritic cells (pDCs), plasma-B cells and T/NK cells. We then averaged across all patients from the five cohorts to define the final pseudo-bulk transcriptomes of these 11 major cell types. Epithelial-specific CMS (eCMS) marker genes were defined as CMS marker genes whose expression was higher in the above-defined epithelial pseudo-bulk transcriptome than in any of the other ten pseudo-bulk transcriptomes. In this manner, we defined 100 eCMS1, 97 eCMS2, 92 eCMS3 and 4 eCMS4 genes. To quantify the expression of eCMS metagenes in epithelial single cells, we zero-centered and scaled each gene (epithelial cells only) in each cohort as before, and then averaged across genes within the same eCMS group to calculate the corresponding eCMS metagene score for each cell. The distributions of single-cell eCMS metagene scores were then calculated for i2 and i3 epithelial cells (Supplementary Fig. 3).

### Enrichment of i2 and i3 markers in chromosomal arms

To quantify enrichment of i2 and i3 markers in specific chromosomal arms, we first defined the set of expressed genes on each arm as those with nonzero expression in at least 5% of cells. We then divided the number of i2 or i3 marker genes by the number of expressed genes. We first performed this analysis on marker genes from the CRC-SG1 cohort (Supplementary Fig. 2d). Subsequently, once epithelial subtypes were defined based on single-cell data from all five cohorts (Fig. 2f), we repeated this chromosomal enrichment analysis (Supplementary Fig. 2e).

### Inferring CNVs from single-cell transcriptomes

We used inferCNV v.1.7.1 to infer CNVs from epithelial single-cell transcriptomes (inferCNV of theTrinity CTAT Project; https://github.com/broadinstitute/inferCNV). The software was provided with raw UMI count data and used at the recommended parameter settings. Thus, we used a cutoff of 0.1 for the minimum average read counts per gene among reference cells, clustered each group of cells separately and denoised our output. For each cohort, normal-like cells were identified as described above (Fig. 2c) and used as the reference for detecting CNVs in tumor cells. The per-gene copy number scores calculated for each cell of each cohort by inferCNV were visualized using v.2.6.2 of the complex heatmap package (Fig. 2d).

### Pseudo-bulk differential expression analysis on epithelial subtypes in five cohorts

To identify the final set of epithelial subtype markers, we used DESeq2 v.1.30.1 to perform differential expression analysis on patient-specific epithelial pseudo-bulk transcriptomes from the five cohorts^74^. In this case, pseudo-bulk transcriptomes were calculated by summing UMI counts across cells, as recommended by DESeq2. For identifying marker genes, we only used primary tumor and adjacent normal data from the 61 of 63 patients whose iCMS classifications based on single-cell transcriptome and copy number profile were consistent. Genes that were detected in fewer than 5% of individuals were discarded and cohort label was defined as a confounding factor. Shrunken log_2_ fold-changes and standard error were estimated using the ‘ashr’ algorithm. Genes with an absolute log_2_ fold-change ≥log_2_(1.5), sequencing depth-normalized mean UMI count ≥75% and adjusted *P* value (Benjamini–Hochberg *Q* value) ≤0.05 were defined as DEGs. DEG analysis was performed in a pairwise manner between each of the three epithelial cell-type pairs (i2 versus i3, i3 versus normal-like, normal-like versus i2). A DEG was defined as ‘Up’ or ‘Down’ in an epithelial cell type only if it was consistently upregulated or downregulated relative to both of the other two cell types. Based on this, we obtained 308 DEGs for i2_Up, 279 for i2_Down, 74 for i3_Up and 54 for i3_Down, totaling 715 iCMS marker genes﻿ (Supplementary Table 14).

### Statistics and reproducibility

Since this was an observational study, the experiments were not randomized. We did not use any statistical method to predetermine cohort size. The researchers were blind to the clinical annotations when they were defining cell and tumor types. Data were only excluded from the analyses if they failed the QC criteria described.

### Reporting summary

Further information on research design is available in the Nature Research Reporting Summary linked to this article.

## Online content

Any methods, additional references, Nature Research reporting summaries, source data, extended data, supplementary information, acknowledgements, peer review information; details of author contributions and competing interests; and statements of data and code availability are available at 10.1038/s41588-022-01100-4.

## Supplementary information

Supplementary Information Supplementary Notes and Figs. 1–12.

Reporting Summary

Supplementary Tables Supplementary Table 1. Summary table of all GSEA and literature curated gene signatures analyzed across iCMS single-cell samples. Two-sided Wilcoxon rank-sum test with no correction was used to calculate all the *P* values. Supplementary Table 2. List of curated GSEA-Hallmarks gene signatures analyzed across iCMS single-cell samples. Supplementary Table 3. List of curated GSEA-KEGG gene signatures analyzed across iCMS single-cell samples. Supplementary Table 4. List of curated mutations related to MAPK gene signatures analyzed across iCMS single-cell samples. Supplementary Table 5. List of curated metabolism gene signatures analyzed across iCMS single-cell samples. Supplementary Table 6. List of curated Wnt/b-catenin pathway gene signatures analyzed across iCMS single-cell samples. Supplementary Table 7. List of curated TGFb pathway gene signatures analyzed across iCMS single-cell samples. Supplementary Table 8. List of curated other cancer-related pathways gene signatures analyzed across iCMS single-cell samples. Supplementary Table 9. List of curated histology and colon cell states gene signatures analyzed across iCMS single-cell samples. Supplementary Table 10. List of curated drug response gene signatures analyzed across iCMS single-cell samples. Supplementary Table 11. Top signaling interactions in NATMI differential signaling analysis. Two-sided Wilcoxon *P* values (with no correction) for testing difference in NATMI expression or specificity scores in i2 (*n* = 9) versus i3 (*n* = 5) in CRC-SG1 cohort are reported. Summary statistics of NATMI expression and specificity scores were computed for i2 and i3 and for comparisons between i2 and i3. Supplementary Table 12. Table summarizing all of the molecular and clinical features described in the study as analyzed over single-cell data and bulk data space. Supplementary Table 13. Patient metadata from five single-cell cohorts. Demographic (sex, age at recruitment), clinical (MSI status, site, sidedness, stage), mutational data (for BRAF, KRAS, TP53, APC, PIK3CA) and iCMS (called by transcriptome and inferCNV) for each patient in the single-cell dataset, and number of samples from LymphNode, Normal and Tumor profiled for each patient. wt, wild type; mut, mutated. Supplementary Table 14. List of all 715 iCMS marker genes (Figure 2F).

## Data Availability

The raw scRNA-seq data are available in the European Genome-phenome Archive (EGA) database with accession codes EGAD00001008555 (CRC-SG1 cohort), EGAD00001008584 (new KUL3 cohort) and EGAD00001008585 (KUL5 cohort). The raw bulk RNA-seq, whole-exome sequencing (WES) and whole-genome sequencing (WGS) data of Singaporean colorectal cancer patients (SG-BULK) are available in the EGA database with accession codes EGAD00001008512 (bulk RNA-seq); EGAD00001008543 (WES); and EGAD00001008566, EGAD00001008574, EGAD00001008592, EGAD00001008625 and EGAD00001008637 (WGS). Processed TPM (SG-BULK) and count expression matrices (scRNA-seq from five cohorts) are available through Synapse under the accession codes syn26720761 (https://www.synapse.org/#!Synapse:syn26720761/) and syn26844071 (https://www.synapse.org/#!Synapse:syn26844071/), ﻿respectively. Published raw scRNA-seq data referenced in the study are available from EGA under the accession codes EGAS00001003779 and EGAS00001003769 (SMC cohort) and from ArrayExpress under the accession codes E-MTAB-8410 and E-MTAB-8412 (KUL3 cohort).

## Code availability

All software used in this study is published and cited either in the main text or Methods. No custom code was used for any aspect of data processing or analysis. Data analysis approaches using published software packages are described in the Methods and Supplementary Notes.
