# Supplementary material for: Single-cell and bulk transcriptome sequencing identifies two epithelial tumor cell states and refines the consensus molecular classification of colorectal cancer
Source: Nat Genet. 2022 Jun 30;54(7):963–75. doi: 10.1038/s41588-022-01100-4 (PMC9279158; doi:10.1038/s41588-022-01100-4)
Supplement: Supplementary file 1 — Supplementary Notes and Figs. 1–12. [file 41588_2022_1100_MOESM1_ESM.pdf]

nature portfolio

https://doi.org/10.1038/s41588-022-01100-4

Supplementary information

Single-cell and bulk transcriptome sequencing identifies two epithelial tumor cell states and refines the consensus molecular classification of colorectal cancer

In the format provided by the authors and unedited

Supplementary Notes

Identifying regulons from scRNA-seq data

We identified regulons from single cell transcriptomes using pySCENIC version 0.10.3[1]. As input, we used the 5-cohort epithelial single cell dataset with cohort-specific zero-centering and scaling. We used GRNboost2 (arboreto 0.1.5) to construct regulatory networks and cisTarget to detect transcription factor binding sites in promoter regions based on motifs from (https://resources.aertslab.org/cistarget/motif2tf/motifs-v9-nr.hgnc-m0.001-o0.0.tbl) and two sets of ranked binding sites: hg38__refseq-r80__500bp_up_and_100bp_down_tss.mc9nr and hg38_refseq-r80_10kb_up_and_down_tss.mc9nr. This approach identified 347 regulons in epithelial cells. The activity level of a regulon in any given cell was then quantified using the AUC metric of pySCENIC.

To cluster cells by regulon activity, we used the regulon x cell matrix of AUC values, after zero- centering and scaling (unit norm) each regulon. We then used PCA for dimensionality reduction (9 PCs) followed by Louvain graph-based clustering as implemented by Seurat to cluster cells based on the entire set of 347 regulons. We then generated patient-specific pseudo-bulk regulomes by averaging across tumor epithelial cells from each patient. To identify differentially active regulomes, we used Seurat's pairwise-DE function to compare pseudo-bulk regulomes from i2 and i3 tumors with the following parameter settings: average of pseudo-bulk AUC across all 61 patients ≥0.01, logfc.threshold ≥ln(1.5), min.pct >0.25, and q-value <0.05.

iCMS metagene score calculation

To further validate the bimodality of our iCMS classification, we then checked the score of iCMS metagene in each individual cell. If the iCMS classification is indeed bimodal, we should be able to find 2 distinct distributions in iCMS2 and iCMS3 metagene score space. Hence, we used the 715 final iCMS marker genes defined above (Fig. 2f), in which, iCMS2 metagene was defined as all genes in iCMS2_up and iCMS3_down, while iCMS3 metagene consists of genes from iCMS3_up and iCMS2_down. To quantify the expression of iCMS metagenes in epithelial single cells, we zero-centered and scaled each gene in each cohort (tumor epithelial cells only), and then averaged across genes within the same iCMS group to calculate the corresponding iCMS metagene score for each cell (Extended data figure 3a).

Hierarchical clustering of epithelial subtypes in differential expression space

To quantify transcriptomic distances between the three genetically defined epithelial cell types, iCMS2-MSS, iCMS3-MSS and iCMS3-MSI, we used DESeq2 as described above to identify DEGs between the the epithelial pseudo-bulk transcriptomes of the corresponding patients. To avoid confounding effects from variation in the number of patients, we selected exactly 12 patients from each of the three groups. This yielded 734 DEGs for i2-MSS vs i3-MSS, 909 for i2-MSS vs i3-MSI, and 63 for i3-MSS vs i3-MSI. Using the number of DEGs as the pairwise distance between any two groups, we then constructed the hierarchical clustering dendrogram (Fig. 2j) using hclust.

Classification and immune signatures analysis from Pelka et al 2021 study

The count matrix of Pelka's dataset was downloaded from NCBI GEO database accession GSE178341. To perform iCMS classification, we first extracted all epithelial cells defined in the original paper (168,295 cells from 62 patients) and then applied our stringent QC cut off as shown in Extended data figure 1b. To reduce batch effects that may be created due to different type of reagent being used in their study (10x 3' v2 vs 10x 3' v3 reagent), we zero-centered and scaled each gene to unit variance (epithelial cells only) within each processed reagent. After that, we performed Louvain graph-based clustering as implemented in Seurat to identify epithelial cell subtypes using the 715 iCMS signatures, which was shown in Fig. 2f, as feature genes. Here, we found 2 distinct epithelial subtypes are again self emerged with some MSS tumors co-mingled with MSI-H tumors (Supplementary Fig. 5a).

To make the final iCMS call for each patient, we then calculate the percentage of cells that were clustered in the opposite group for each patient (Supplementary Fig. 5b). If a patient has more than 10% of their cells clustered together in the opposite group, then that patient will be called as indeterminate (patient inside the red boxes in Supplementary Fig. 5b). Furthermore, we also discarded patient C162 from our downstream analysis because this patient was processed with both 10x 3' v2 and 10x 3' v3 reagent (patient inside the blue boxes in Supplementary Fig. 5b). In the end, 11 patients were classified as iCMS2, 44 patients were classified as iCMS3, and 6 patients were classified as indeterminate.

To calculate T cell program activity scores in TCGA bulk data, we used the AddModuleScore function of Seurat v4[2], using the program-specific gene signatures from Pelka et al[3]

iCMS classification of tumors based on bulk transcriptome

We compiled 15 sets of bulk tumor transcriptomes and analyzed each set individually to assign tumors to one of the following three classes: iCMS2, iCMS3, indeterminate. First, the FPKM/TPM/fRMA expression values in a dataset were log2-transformed and then each gene was standardized to obtain expression z-scores. The resulting standardized bulk expression matrix was used for classification by the nearest template prediction (NTP) algorithm[4], as implemented in the CMScaller package previously used for CRIS subtype classification [https://github.com/peterawe/CMScaller/]. Only the 715 iCMS marker genes described above were used in this analysis. To construct the template z-score vector for iCMS2 bulk transcriptomes, i2_Up and i3_Down markers were assigned a value of +1 and i3_Up and i2_Down markers were assigned a value of -1. The same z-score vector, with opposite sign, was used as the template for iCMS3. Each sample was classified by NTP as iCMS2 or iCMS3 if its distance to the corresponding template was significantly lower (FDR<0.05) than that of 1,000 random permutations of the gene labels. Statistical significance was estimated only for the template nearest to each sample and samples failing the FDR cutoff were defined as indeterminate. Associations between bulk tumor iCMS2 and iCMS3 intrinsic epithelial status and clinico-molecular features were analyzed. For each association with a clinical or molecular parameter, the patients evaluated included all those with a determined iCMS status and for which the annotation for the specific clinical or molecular feature of interest was available.

Co-expression analysis of bulk tumor transcriptomes

Co-expression analysis and tumor clustering were performed on the RMA-normalized bulk-tumor microarray expression datasets from 12 of the 13 CMS cohorts, plus the TCGA and SG-Bulk RNA-seq datasets (see above for accession details). The PETACC3 cohort was excluded since the number of interrogated genes was relatively small. In Affymetrix data, probesets with the same gene symbol were combined by averaging. Mean centering in logarithmic space was performed for each gene in each cohort, prior to combining into a single data matrix. Genes with low expression variability (with SD less than 0.4 in log2 scale) were removed. A final gene expression matrix of 12,164 genes and 2,873 samples was obtained after removing data from normal tissues. Samples were hierarchically clustered using Ward's method and genes using average linkage. Computation and visualization were performed using the R package nclust (https://gitlab.com/pwirapati/nclust).

Survival analyses

Survival analysis was performed on the Guinney[5]and SG-Bulk cohorts. For the TCGA cohort, updated outcome data were obtained from Liu et al[6]. For relapsed patients, survival after relapse (SAR) was inferred using the difference between overall survival time and time to relapse. Survival analysis was performed using the R package "survival" (https://github.com/therneau/survival). Hazard ratios and statistical tests were performed using the Cox proportional-hazards method and stratified by cohorts. Survival curves were estimated using the Kaplan-Meier method.

Statistical analysis of DNA mutations

Comparisons of the frequency of mutations between groups of iCMS2 and iCMS3, MSS and MSI, iCMS2-MSS and iCMS3-MSS, as well as iCMS3-MSS and iCMS3-MSI were performed using two-sided Fisher's exact test, with Benjamini-Hochberg correction. *APC* mutations were further analysed by position, where the cumulative frequencies of truncating mutations were compared between iCMS2 vs iCMS3, iCMS2_MSS vs iCMS3_MSS vs iCMS3_MSI, as well as iCMS2_MSS_NF vs iCMS2_MSS_F vs iCMS3_MSS_NF vs iCMS3_MSS_F vs iCMS3_MSI. For the pairwise comparison, statistical significance of positional differences in APC mutations was evaluated using the Kolmogorov–Smirnov test (Extended data figure 6c).

Gene set enrichment analysis of iCMS2 vs iCMS3

We used DESeq2 as described above to score differential expression between iCMS2 and iCMS3 epithelial pseudo-bulk transcriptomes (57 patients, tumor samples only). Genes with average expression within the lowest quartile were discarded and then the differential expression score was defined for each remaining gene as -log10(p-value)*log2(fold change) and the genes were then ranked by this score. Systematic differences between iCMS2 and iCMS3 epithelial cells were then identified using the pre-ranked gene set enrichment analysis (GSEA) method implemented in GSEA software v4.1.0[7](enrichment statistics: "classic", 1000 permutations). Enrichment was evaluated in gene sets from the Molecular Signatures Database (MSigDB) (Fig. 4e and Supplementary Fig. 7) and from Guinney et al. 2015 paper (Fig. 7e).

Gene set enrichment analysis of 5 CRC subtypes based on bulk transcriptomes

We also used the method described above to identify gene sets specifically expressed in each of the 5 tumor subtypes, based on bulk transcriptomes from TCGA. In this case, we performed GSEA on differential gene expression scores from the following 5 comparisons: iCMS2_MSS_NF vs. all, iCMS2_MSS_F vs. all, iCMS3_MSI vs. all, iCMS3_MSS_NF vs. all, iCMS3_MSS_F vs. all. After discarding pathways with FDR q-val > 0.05, leading-edge genes (genes that contributed to the observed enrichment) for each pathway were combined across the 5 sets of GSEA results. The mean z-score of the combined leading-edge genes for a pathway was defined as the pathway activity score of a tumor.

Differential methylation analyses

To identify differentially methylated CpG sites between the iCMS2 and iCMS3 subtypes, we utilized 176 colorectal adenocarcinoma samples of TCGA Infinium HumanMethylation27 BeadChip array data with the corresponding CIMP annotations. We applied t-test and an average methylation difference threshold of 0.2 between the two groups to select the informative CpG sites. Using this approach, we identified 978 CpG sites that were differentially methylated in iCMS2 and iCMS3 subtypes. The methylation values of the CpG sites were visualized using a heatmap and corresponding phenotypic properties of the samples were annotated.

Finding differentially expressed genes for each major cell type

To identify the cell type specific marker for each major cell type, we used DESeq2 version 1.30.1 to perform differential expression analysis on patient-specific epithelial pseudo-bulk transcriptomes from CRC-SG1 cohort[8]. Here, we first summed UMI count to define the patient- specific pseudo-bulk of B, endothelial, epithelial, fibroblast, mast, McDC, neutrophils, Plasma-B and T/NK cells. Genes that were detected in fewer than 5% of individuals were discarded.

Shrunken log2 fold changes (LFC) and standard error (SE) were estimated using the "ashr" algorithm. Genes with an absolute LFC greater ≥log2(1.5), sequencing depth-normalized mean UMI count ≥75% percentile and adjusted p-value (Benjamini–Hochberg q-value) ≤0.05 were defined as DEGs. DEG analysis was performed in a pairwise manner between each of the 9 major cell type pairs (Ex: B vs.

Endothelial; B vs. fibroblast; B vs. epithelial, etc), and a gene will be called cell type specific markers if it was consistently upregulated relative to 8 other cell types (pDC and entericglial was excluded). Here, we obtained: B = 120, endothelial = 200, epithelial = 259, fibroblast = 242, mast = 115, McDC = 113, neutrophils = 285, plasma_B = 457, and T_NK = 84 marker genes. After that, we calculated a metagene score for each cell type specific marker by averaging all marker genes in their respective group for each individual patient. The metagene scores for each cell type were then visualized using complexheatmap package for 577 bulk tumor transcriptomes (Fig. 6a).

Finding differentially expressed genes between iCMS2-MSS-F and iCMS3-MSS-F

We performed DESeq2 version 1.30.1 on TCGA bulk transcriptomes. Genes that were detected less than 10 counts in whole samples were discarded. Shrunken log2 fold changes (LFC) and standard error (SE) were calculated using the "ashr" algorithm. Genes with an sequencing depth- normalized mean UMI count ≥75% percentile, absolute LFC greater ≥log2(2), and adjusted p- value (Benjamini–Hochberg q-value) ≤0.01 were defined as differentially expressed. Furthermore, to map bulk RNA-seq derived differentially expressed genes on single cell space, we constructed pseudo-bulk expression matrices by cell type from CRC-SG1 single cell cohort using Seurat's AverageExpression() function .

Signaling analysis

We used the scRNA-seq data from the CRC-SG1 cohort to infer signaling interactions between cell types in CRC tumors. For greater resolution of TME components, we further sub-clustered T/NK cells and fibroblasts using the clustering procedure described above for CRC-SG1 using the following resolution parameters: Fibroblast 1st round: 0.4, 2nd round: 0.55; T cells 1st round: 0.25, 2nd round: 0.55. Mesenchymal cells were then annotated as 6 broad groups: CAF subtypes A and B, proliferating CAFs, smooth muscle cells (SM), normal fibroblasts and other (unclassified) fibroblasts, while T cells were grouped into CD4, CD8 and innate subsets. We then selected cells from primary tumor samples, and removed normal epithelial and normal fibroblast cells. This resulted in a high-quality dataset of n = 124,857 cells classified into 13 cell types: the above- mentioned fibroblast and T/NK cell subtypes, plus epithelial, B, plasmaB, myeloid (McDC) and endothelial cells.

To infer signaling interactions, we conducted NicheNet (v1.0.0)[9]ligand activity analysis using the i2 up, i2 down, i3 up and i3 down gene sets. A gene was determined to be expressed in a cell type if it was expressed in at least 10% of cells in that cell type in the CRC-SG1 cohort. Ligands were then ranked based on their ligand activity using the Pearson correlation coefficient, and target genes of each ligand were defined as the top 200 most strongly predicted targets ranked by regulatory potential score. For visualization of regulatory potential scores, a quantile cutoff of 0.33 was used. Finally, to determine possible sources of top-ranked ligands in the tumor microenvironment, we visualized the average scaled patient-wise pseudobulk expression of each ligand in each cell type across patients in the CRC-SG1 cohort.

In addition, we also used NATMI[10] to infer interactions between ligands from each of the 16 possible sender cell types and receptors from each of the 16 possible receiver types. For each patient, NATMI's (git commit: 3ef1f05) `ExtractEdges.py` was used to compute expression and specificity scores of each interaction edge, defined by a sender cell type, receiver cell type, ligand and receptor. Then, for each interaction edge, the two-sided Wilcoxon rank-sum test was used to test the difference in the expression and specificity scores between i2 and i3 samples. We reasoned that top differential interactions should be relatively specific, and that the ligands and receptors in the condition with the higher score should be expressed at non-negligible levels by the sender and receiver cell types respectively. Hence to prioritize differential interactions, we applied the following filters: absolute difference in specificity score>0.025, specificity score in condition with the higher score>0.05, average fraction of ligand expressed in samples in the condition with the higher score>0.1, average fraction of receptor expressed in samples in the condition with the higher score>0.1, Wilcoxon p-value testing difference in expression or specificity score<0.05. Then, to focus on top differential interactions involving tumor epithelial cells, we further filtered interactions so that either the sender cell is tumor epithelial and the ligand is differentially expressed in i2 vs. i3, or the receiver cell is tumor epithelial and the receptor is differentially expressed in i2 vs. i3. This resulted in a total of 33 top interactions between i2 and i3 (Supplementary Table 2). To further inspect ligand-receptor interactions of

interest, we visualized average scaled patient-wise pseudo-bulk expression of the ligand and receptor in putative sender and receiver cell types across patients in the CRC-SG1 cohort using dotplots.

Associations between iCMS and polyp subtypes

Recently, Chen *et al.* reported that potentially malignant colorectal polyps could be divided into two groups, adenomas (ADs) and sessile serrated lesions (SSLs). To identify potential relationships between the two iCMS subtypes and these two polyp subtypes, we extracted all polyp marker genes defined in Fig. 3a and b from Chen et al[11]. We then examined the expression of these polyp markers in iCMS2, iCMS3 and normal-like epithelial cells from the 61 patients across 5 cohorts. The mean iCMS2 transcriptome was calculated by averaging the 38 corresponding patient-specific epithelial pseudo-bulk transcriptomes. Mean iCMS3 and normal-like epithelial transcriptomes were calculated in a similar manner by averaging across patients.

Tissue enrichment analysis

Tissue enrichment analysis was performed using the TissueEnrich package (version 1.10.1)[12]. in this software, tissue-specific genes were defined by processing RNA-Seq data from the Human Protein Atlas (HPA)[13]GTEx[14]and mouse ENCODE[15]using the algorithm from the HPA[13]After that, a hypergeometric test will be used to determine if the tissue-specific genes are enriched among the input genes[12]. For our analysis, we used the 715 iCMS marker genes as input genes and defined all expressed genes (at least expressed in more than 5% of total cells) as a background geneset. We then followed all steps which were described in the package vignettes. All default parameters were used.

Drug response analysis

The drug sensitivity data (IC50) was extracted from CTRPv2 database[16](Cancer Therapeutics Response Portal (broadinstitute.org)). To classify cell lines, we first extracted transcriptomic information of commercial colon cell lines from relevant databases[17]and performed iCMS classification using our method described above for the bulk datasets. Across the cohort, 16 lines were classified as iCMS2, 23 for iCMS3 and 5 were indeterminate. We then generated box plots comparing drug sensitivity (Log10 (IC50)) across iCMS2 versus iCMS3 cell lines for each of the 477 drugs tested and showed 3 selected drugs which were found to be statistically different ($p<0.05$) for iCMS2 and iCMS3. Statistical comparison of drug sensitivity between the two groups (iCMS2 and iCMS3) was performed by a two-sided Wilcoxon rank-sum test for each drug.

Supplementary Figures

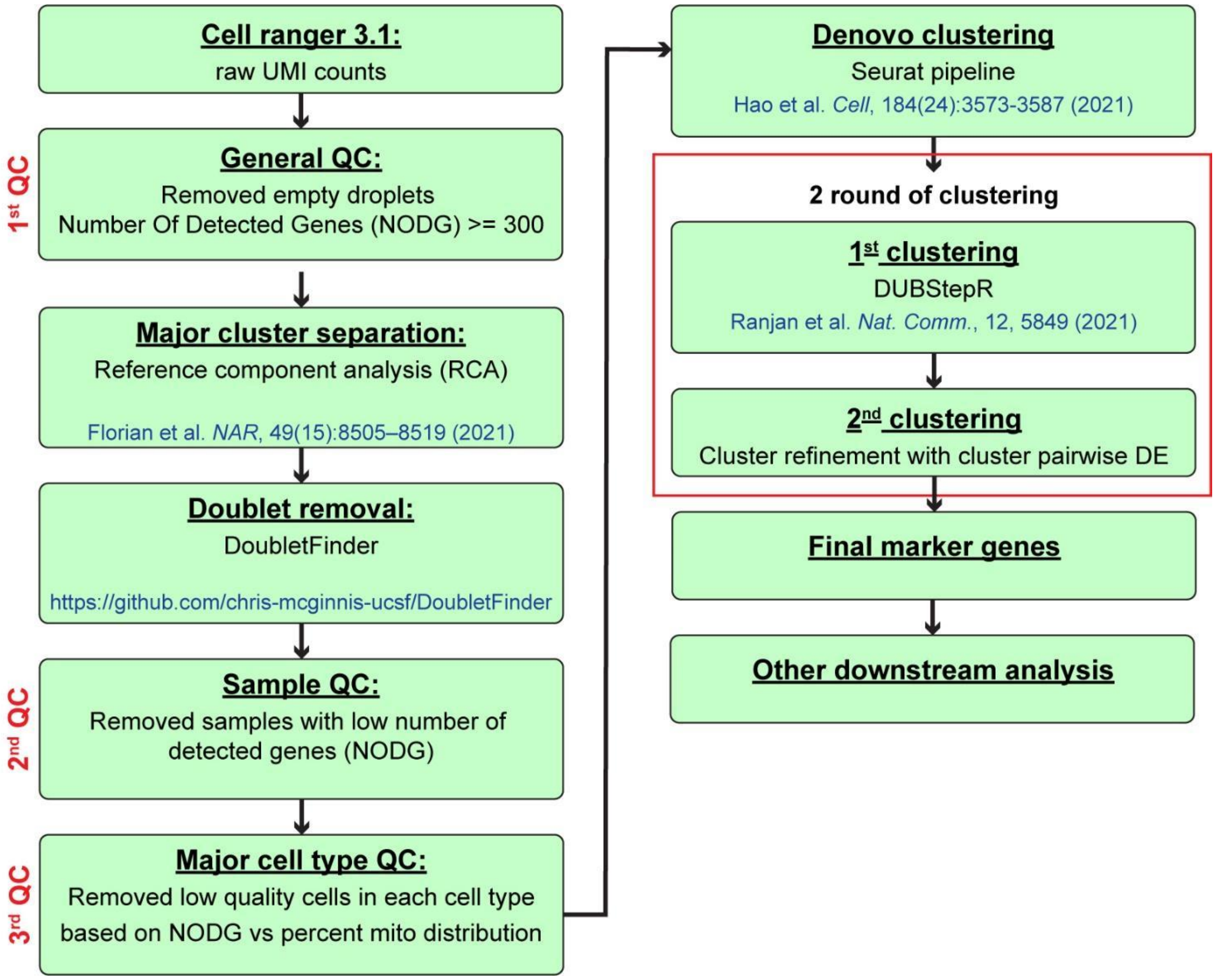

Supplementary Figure 1: Flowchart of the analysis pipeline used in this study.

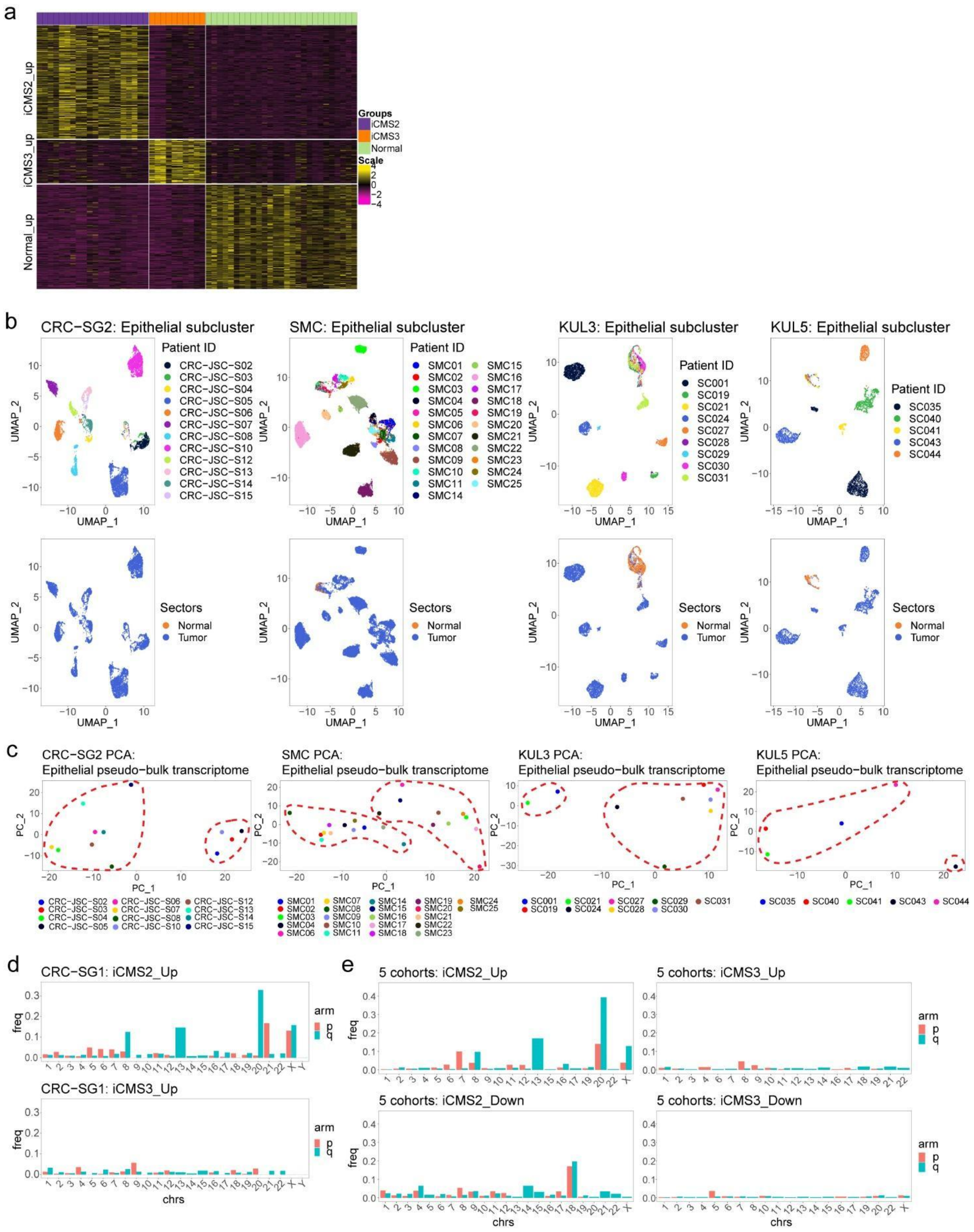

Supplementary Figure 2: Sub-clustering of epithelial cell from 4 independent cohorts

a. Heatmap of differentially expressed genes (n=848) in sample-specific pseudo-bulk, colored by averaged scaled gene expression in CRC-SG1 (patients = 14). The columns were arranged by iCMS subtype (iCMS2, iCMS3 and Normal-like), while the rows were grouped by DEGs type. (iCMS2_Up: 368 genes; iCMS3_Up: 141 genes; normal_Up: 339 genes). **b.** UMAP of epithelial cells colored by (upper panel) patient ID and (lower panel) tumor sectors in each respective cohort (CRC-SG2 = 8,744, SMC = 15,570, KUL3 = 5,582, and KUL5 = 3,339 cells). **c.** PCA of epithelial patient specific pseudo-bulk transcriptome for each respective cohort (CRC-SG2 = 12, SMC = 23, KUL3 = 9, and KUL5 = 5 patients). (**d,e**). Barplot showing the fraction of differentially expressed genes in each chromosomal arms for (**d**) CRC-SG1 DEGs (n=848) and (**e**) 5 cohorts DEGs (n=715).

	B	Endothelial	Entericglial	Epithelial	Fibroblast	Mast	McDC	Neutrophils	pDC	Plasma-B	T/NK	Total
CMS1	4	26	14	100	19	6	13	3	12	2	16	215
CMS2	1	4	3	97	8	6	1	0	2	0	0	122
CMS3	1	5	7	92	8	9	1	1	8	0	3	135
CMS4	2	26	20	4	126	1	7	2	5	0	1	194
Total	8	61	44	293	161	22	22	6	27	2	20	666

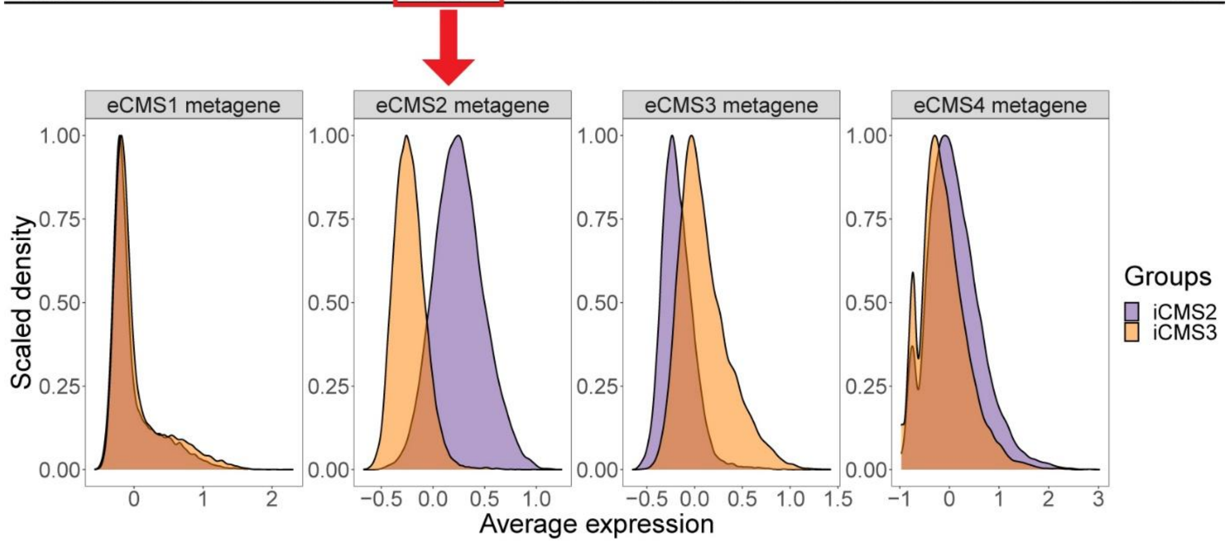

Supplementary Figure 3: Association between iCMS and CMS classification for epithelial subtypes based on bulk transcriptomes.

(upper panel) Table listing where each CMS marker is expressed inside the tumour scRNA-seq data from 5 cohorts. Epithelial-specific CMS (eCMS) genes were defined as genes whose expression was higher in epithelial pseudo-bulk transcriptomes than other 10 pseudo-bulk transcriptomes. (lower panel) Histogram showing the expression of eCMS metagenes in epithelial single cells. The metagene scores for each cell were calculated by averaging the scaled expressions within the same eCMS group.

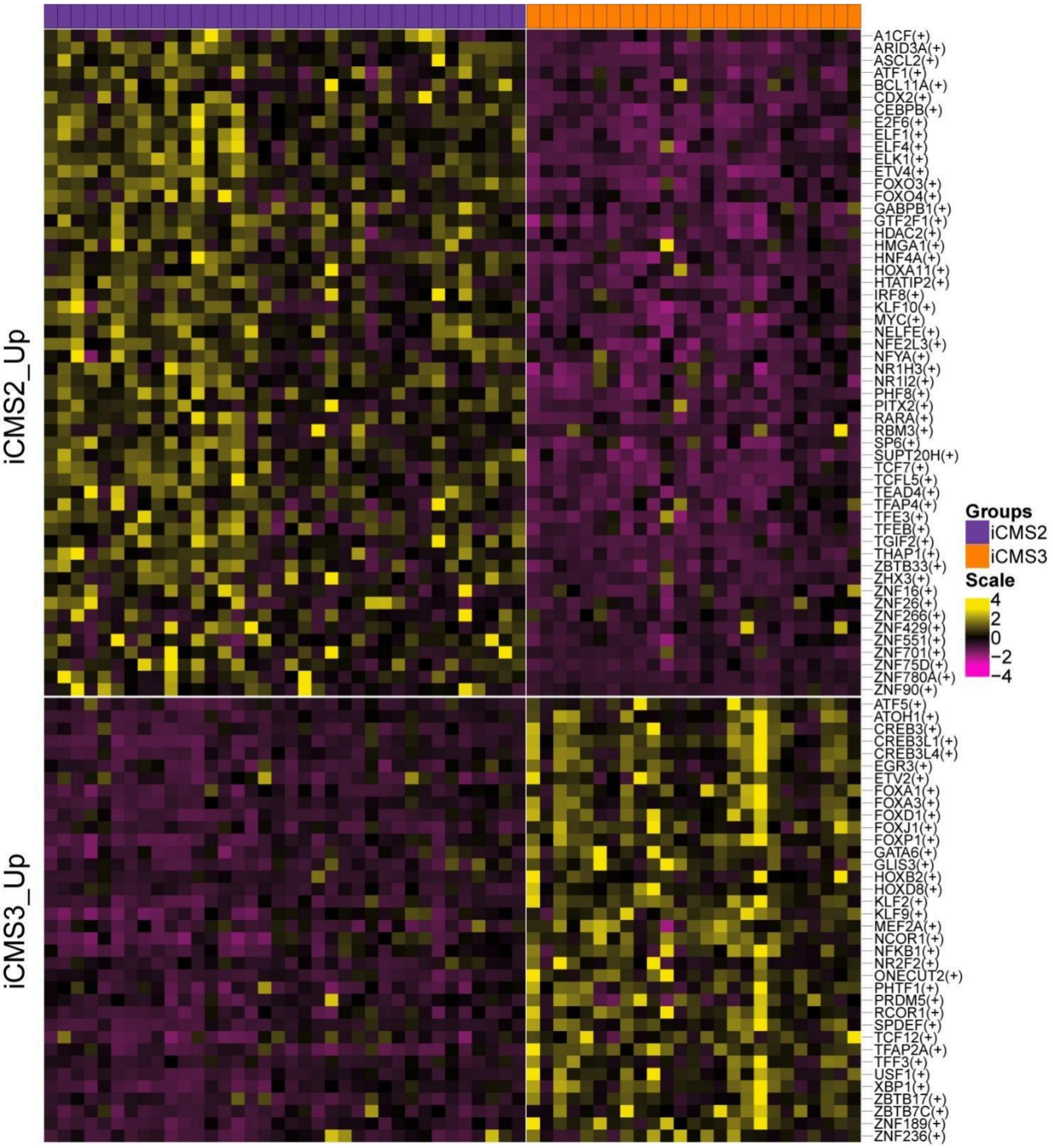

Supplementary Figure 4: Heatmap of differentially expressed regulons (n=90 genes) in patient-specific pseudo-bulk (n=61 patients), colored by scaled regulon activity score (AUC score) obtained from SCENIC analysis. The columns were arranged by iCMS subtype (iCMS2 and iCMS3), while the rows were grouped by DE type (iCMS2 Up = 54 and iCMS3 Up = 36).

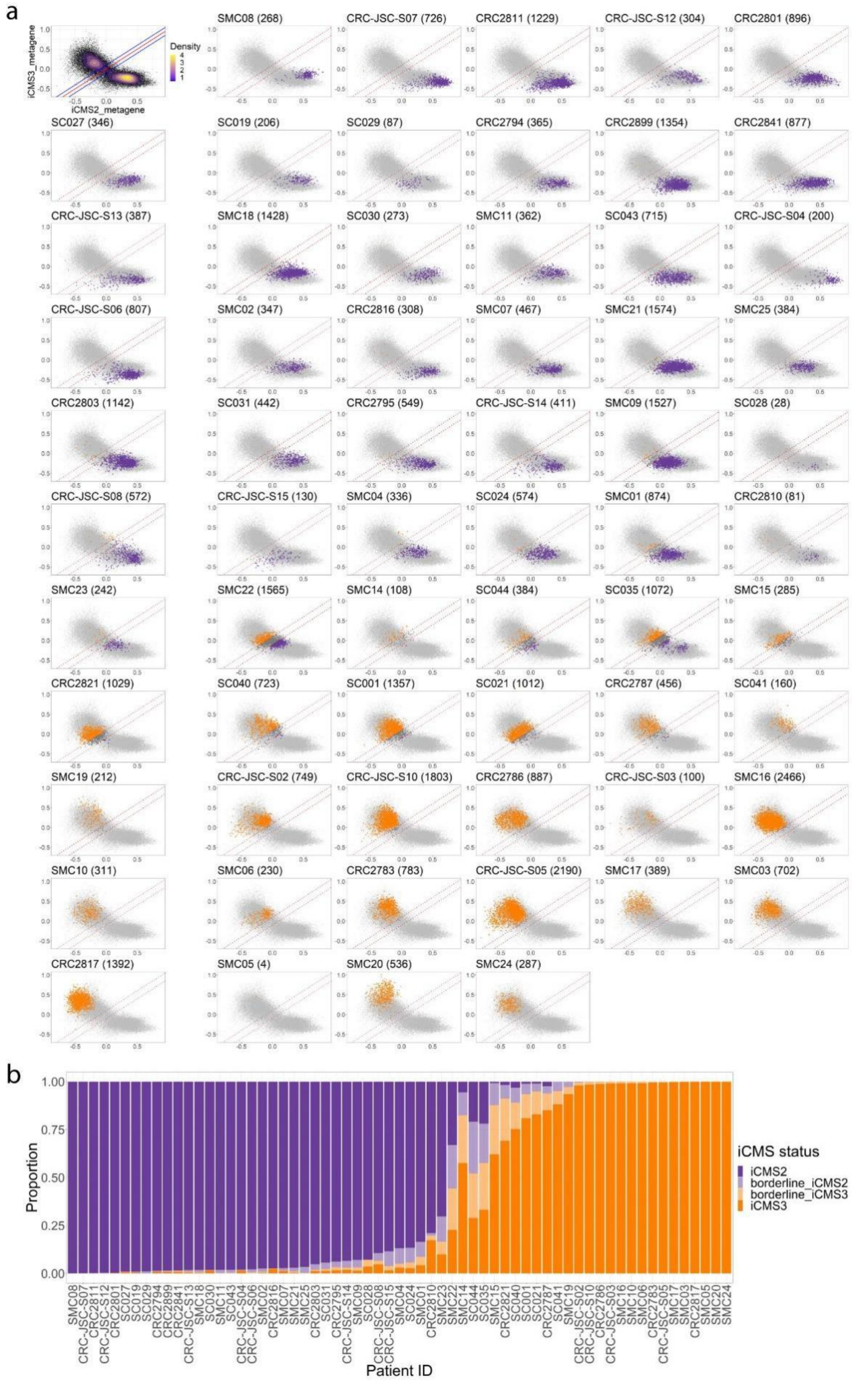

Supplementary Figure 5: Hybrid tumors are less common in iCMS subtype

a. Scatterplot of 42,010 tumor epithelial cells from 5 cohorts in iCMS metagene space. X-axis: iCMS2 metagene score; Y-axis: iCMS3 metagene score. The mode at the bottom right of the scatterplot corresponds to iCMS2 cells, while the opposite mode corresponds to iCMS3. In the top left panel, the solid red line indicates (x-y)=0, while the solid blue lines indicate |x-y|=0.1. In the remaining panels, the dashed red lines indicate |x-y|=0.1. Cells lying in between the dashed red lines are defined as borderline iCMS2 (x-y>0) or borderline iCMS3 (x-y<0). **b.** Barplot representing the proportion of cells from each of the four categories in the 63 subjects.

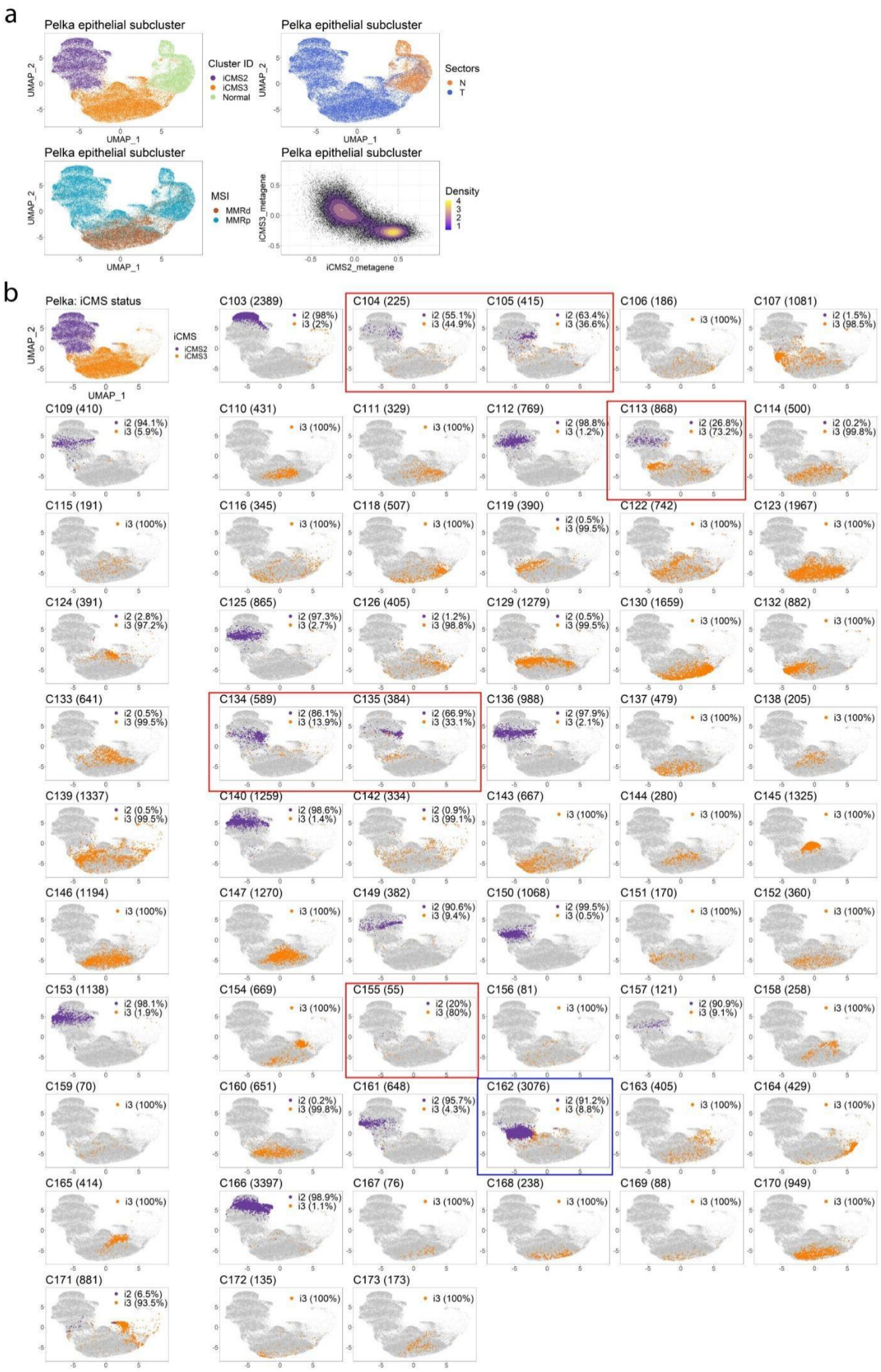

Supplementary Figure 6: iCMS classification of Pelka's dataset

a. UMAP visualization of 56,551 epithelial cells from Pelka's dataset in transcriptomic space colored by iCMS label from denovo clustering (top left), tumor sectors (top right), and MSI status (bottom left).

(bottom right) Density plot of 44,110 tumor epithelial cells from Pelka's dataset in iCMS metagene space. The mode at the bottom right of the scatterplot corresponds to iCMS2 cells, while the opposite mode corresponds to iCMS3. **b.** UMAP visualization of tumor epithelial cells from Pelka's dataset in transcriptomic space, grouped by patients, and colored by iCMS label. The numbers next to patient ID in each plot indicates the total number of tumor epithelial cells for that particular patient. The percentage on the bottom right of the UMAP indicates the number of cells that were clustered in i2 clusters (purple color) or i3 clusters (orange color). Patients inside the red box were labelled as indeterminate because it has more than 10% of their cells clustered together in the opposite group. The patient inside the blue box was discarded downstream because it is the only patient that was processed with both 10x 3' v2 and 10x 3' v3 reagent. In the end, 11 patients were classified as iCMS2, 44 patients were classified as iCMS3, and 6 patients were classified as indeterminate.

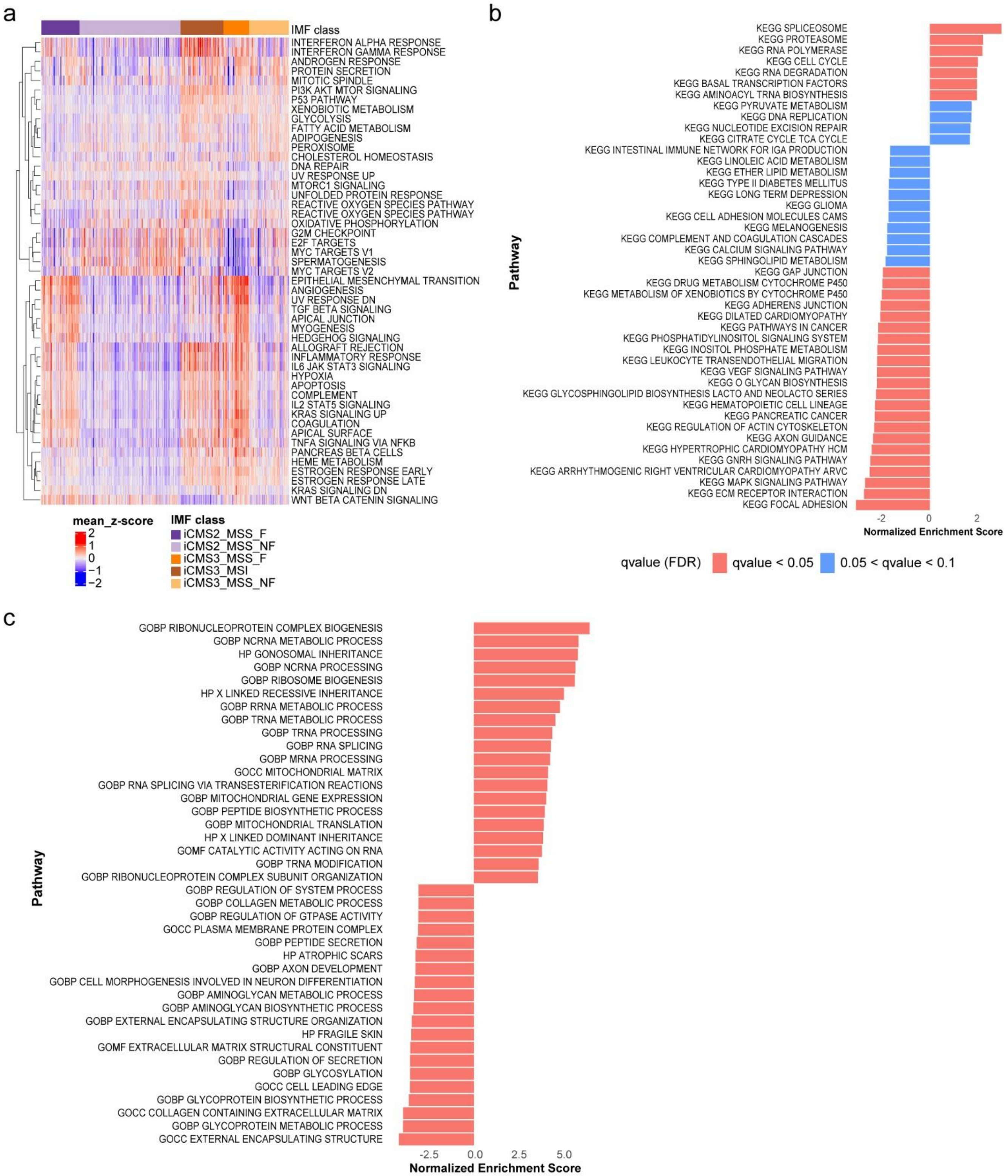

Supplementary Figure 7: GSEA results of several pathways in iCMS2 versus iCMS3.

a. 5-way gene set enrichment analysis (GSEA). The heatmap shows pathway activity scores of TCGA bulk tumors (n= 462) for MSigDB hallmark pathways. Activity scores are calculated based on combined leading edge genes from 5 sets of GSEA, each specific to an IMF group, performed on TCGA bulk transcriptomes. **b.** GSEA results of MSigDB KEGG pathways in iCMS2 vs. iCMS3. X-axis: normalised enrichment score (NES) in iCMS2 relative to iCMS3. **c.** Gene set enrichment analysis (GSEA) results of MSigDB Gene Ontology (GO) pathways in iCMS2 vs. iCMS3. X-axis: NES in iCMS2 relative to iCMS3.

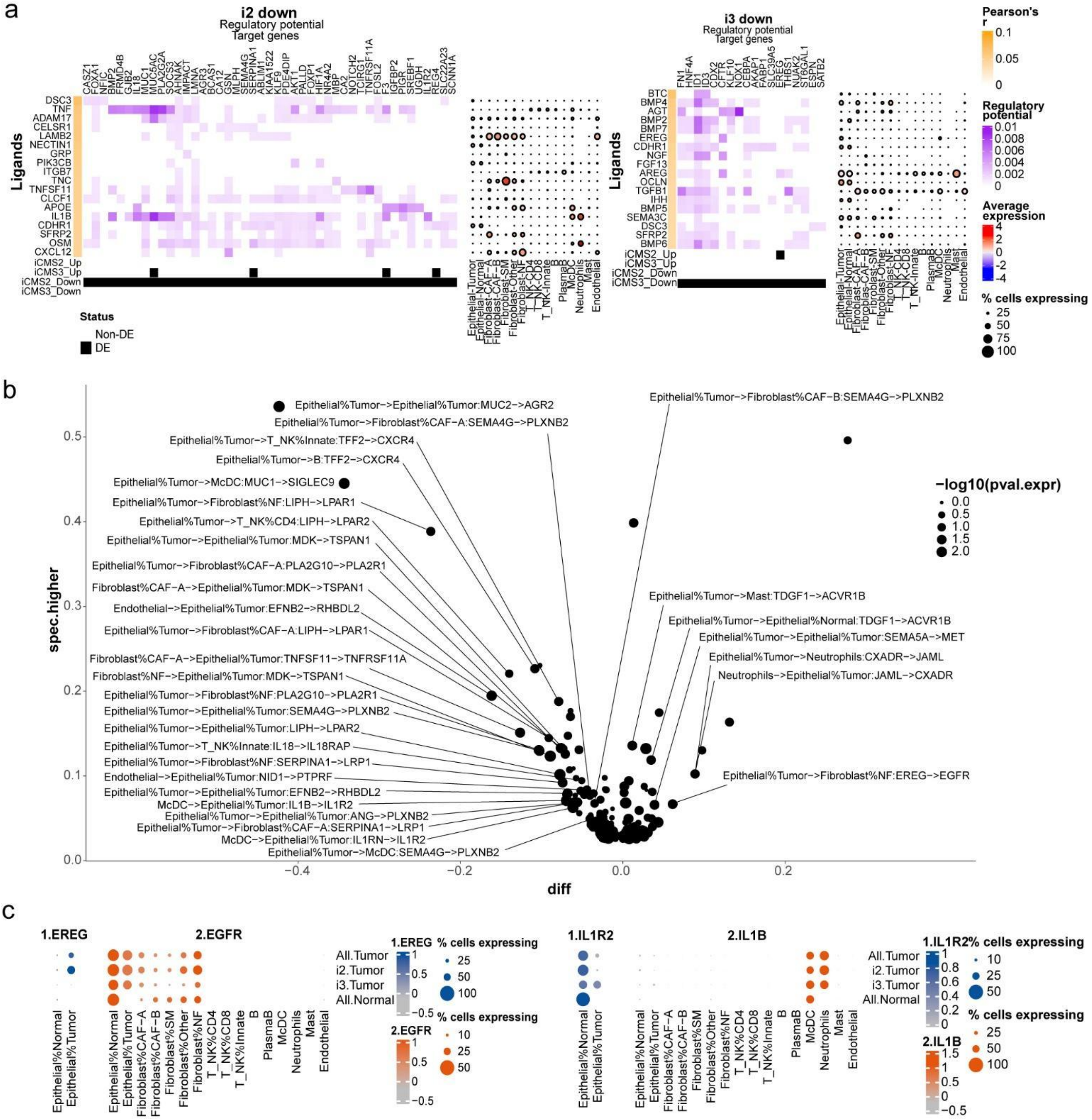

Supplementary Figure 8: Differential signalling interactions between tumor epithelial cells and the tumor microenvironment.

a. Results of NicheNet analysis for i3 up (left) and i3 down (right). For each gene set, the heatmap on the left depicts the regulatory potential scores (purple) for the top 200 target genes of each of the top 20 ligands ranked by Pearson correlation (orange) after filtering at a quantile cutoff of 0.33 for the regulatory potential score. The dotplot on the right depicts the average scaled patient-wise psuedobulk expression of each of the top-ranked ligands for each cell type across patients in the CRC-SG1 cohort. Dot size corresponds to the percent of cells expressing the ligand in each cell type. **b.** Prioritization of top differential interactions (labeled in plot, Table S2) in NATMI analysis via filtering on the difference in specificity score between i2 and i3, as well as the specificity score in the condition with the higher score. Dot size and color correspond to unadjusted two-sided Wilcoxon p-value testing for difference in expression and specificity scores respectively (i2, n = 9 vs. i3, n = 5). **c.** Dotplots depicting the average scaled patient-wise pseudo-bulk expression of ligand and receptor pairs of interest in putative sender and receiver cell types across patients in the CRC-SG1 cohort. Dot size corresponds to the percent of cells expressing the ligand and receptor in the respective cell types.

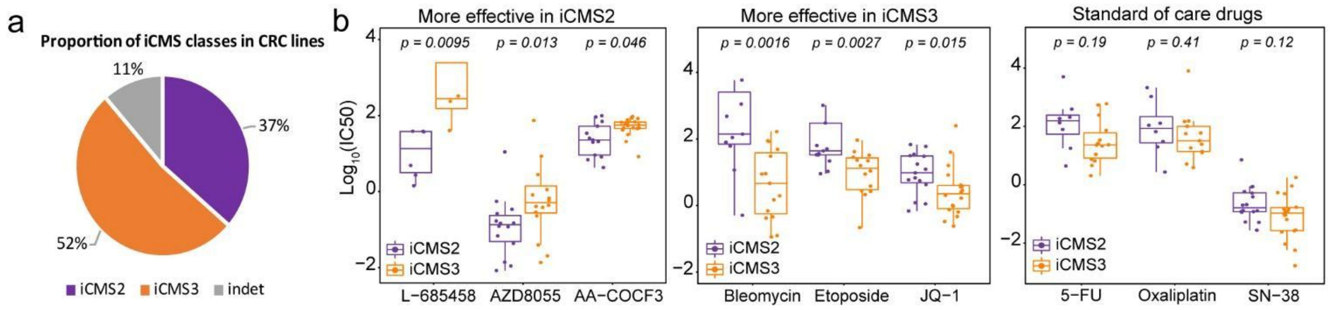

Supplementary Figure 9: Analysis of differential drug response in iCMS cell lines

a. Pie Chart showing proportion of different iCMS classes in commercial cell lines. **b.** Selected drugs more effective in iCMS3 cell lines (Bleomycin, JQ-1 and Etoposide), iCMS2 cell lines (L-685458, AZD-8055, AA-COCF3) and across standard-of-care treatments (5-FU, Oxaliplatin and SN-38). Drug efficacies are represented by logarithm base 10 of half the maximal inhibitory concentration (Log10(IC50)). All p values for box plots are calculated from a two-sided Wilcoxon rank-sum test. Center line indicate the median, and box edges indicate the 25th (Q1) and 75th (Q3) percentiles. Whiskers are plotted at 1.5xIQR and data beyond the end of the whisker are outliers.

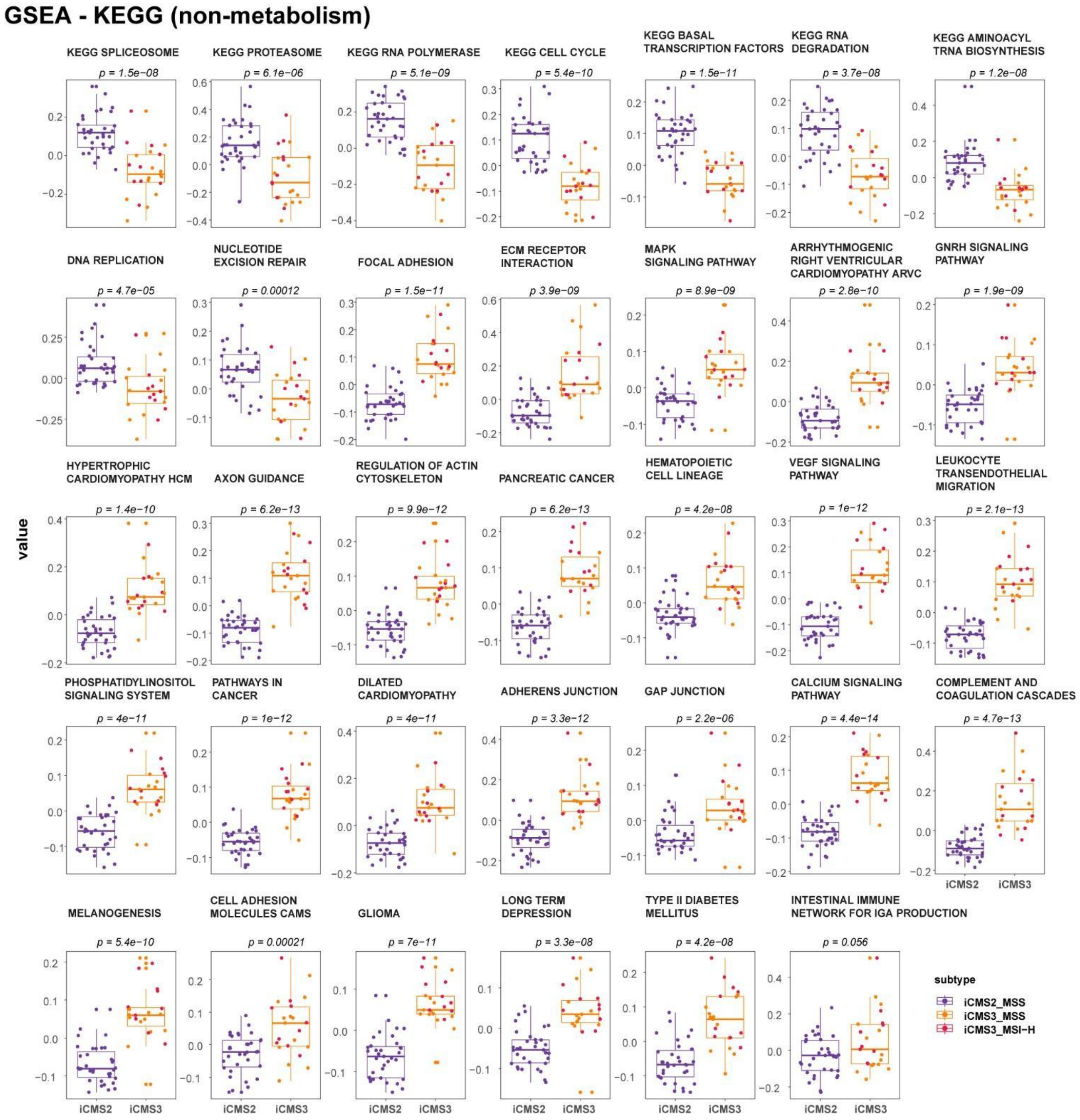

Supplementary Figure 10: Comparison of metagene signature of selected GSEA-KEGG (non-metabolic) pathways in single cell epithelial cells across iCMS classes

Box plots of metagene scores comparing iCMS2 (n=35) versus iCMS3 (n=23) in patient-specific pseudo-bulk. Within the iCMS3 group, i3-MSI (n=10) samples are labelled by red jitter points and i3-MSS samples are labelled by orange jitter points. The metagene scores for each patient pseudo-bulk was calculated by averaging the scaled expressions of all genes in the geneset in the given patient. P-values were calculated by two-sided Wilcoxon rank-sum test. Center line indicate the median, and box edges indicate the 25th (Q1) and 75th (Q3) percentiles. Whiskers are plotted at 1.5xIQR and data beyond the end of the whisker are outliers.

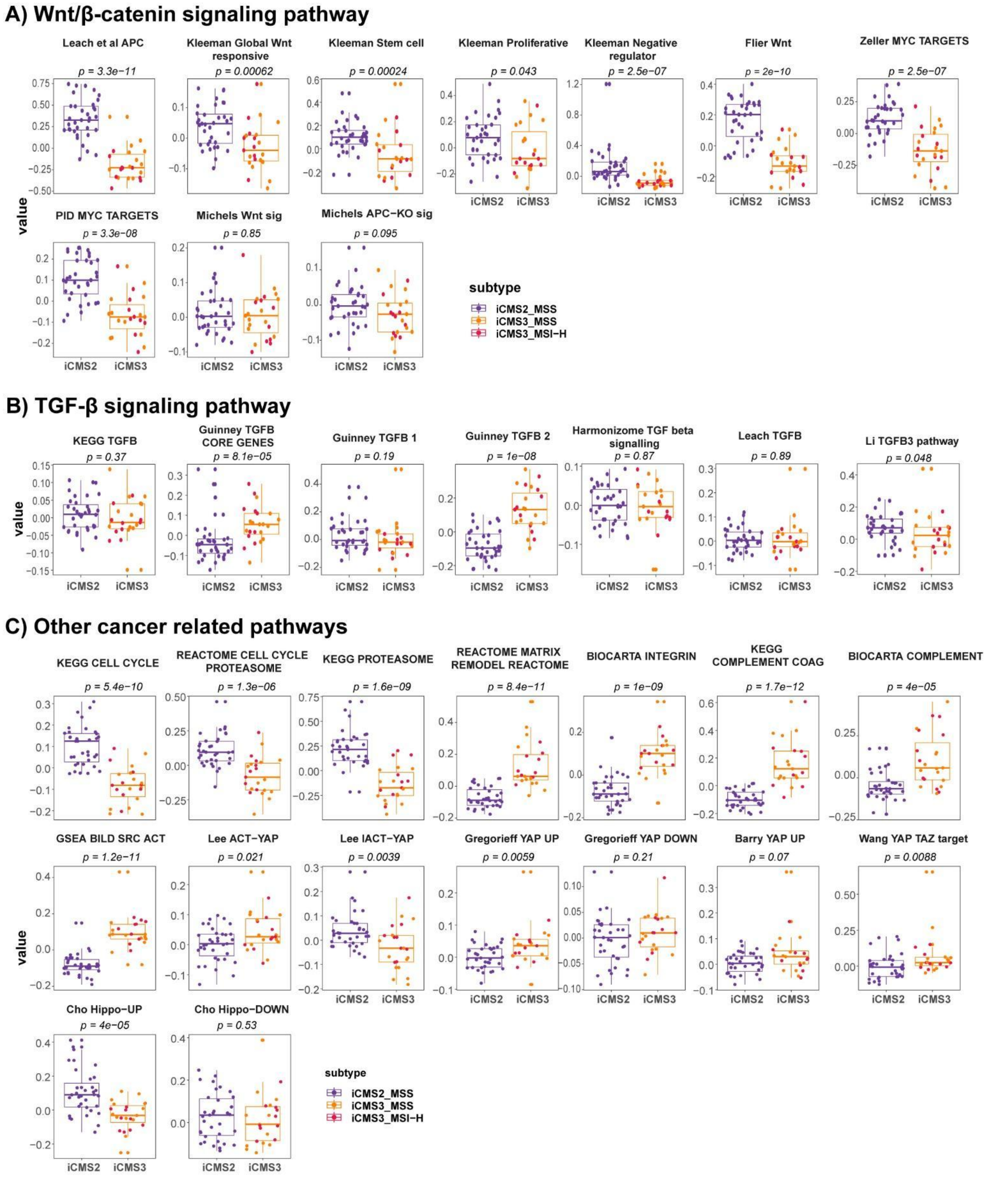

Supplementary Figure 11: Comparison of metagene signature of selected Wnt/β-catenin pathway (A), TGF-β pathway (B), and other cancer-related pathways (C) in single cell epithelial cells across iCMS classes

Box plots of metagene scores comparing iCMS2 (n=35) versus iCMS3 (n=23) in patient-specific pseudo-bulk. Within the iCMS3 group, i3-MSI (n=10) samples are labelled by red jitter points and i3-MSS samples are labelled by orange jitter points. The metagene scores for each patient pseudo-bulk was calculated by averaging the scaled expressions of all genes in the geneset in the given patient. P-values were calculated by two-sided Wilcoxon rank-sum test. Center line indicate the median, and box edges indicate the 25th (Q1) and 75th (Q3) percentiles. Whiskers are plotted at 1.5xIQR and data beyond the end of the whisker are outliers.

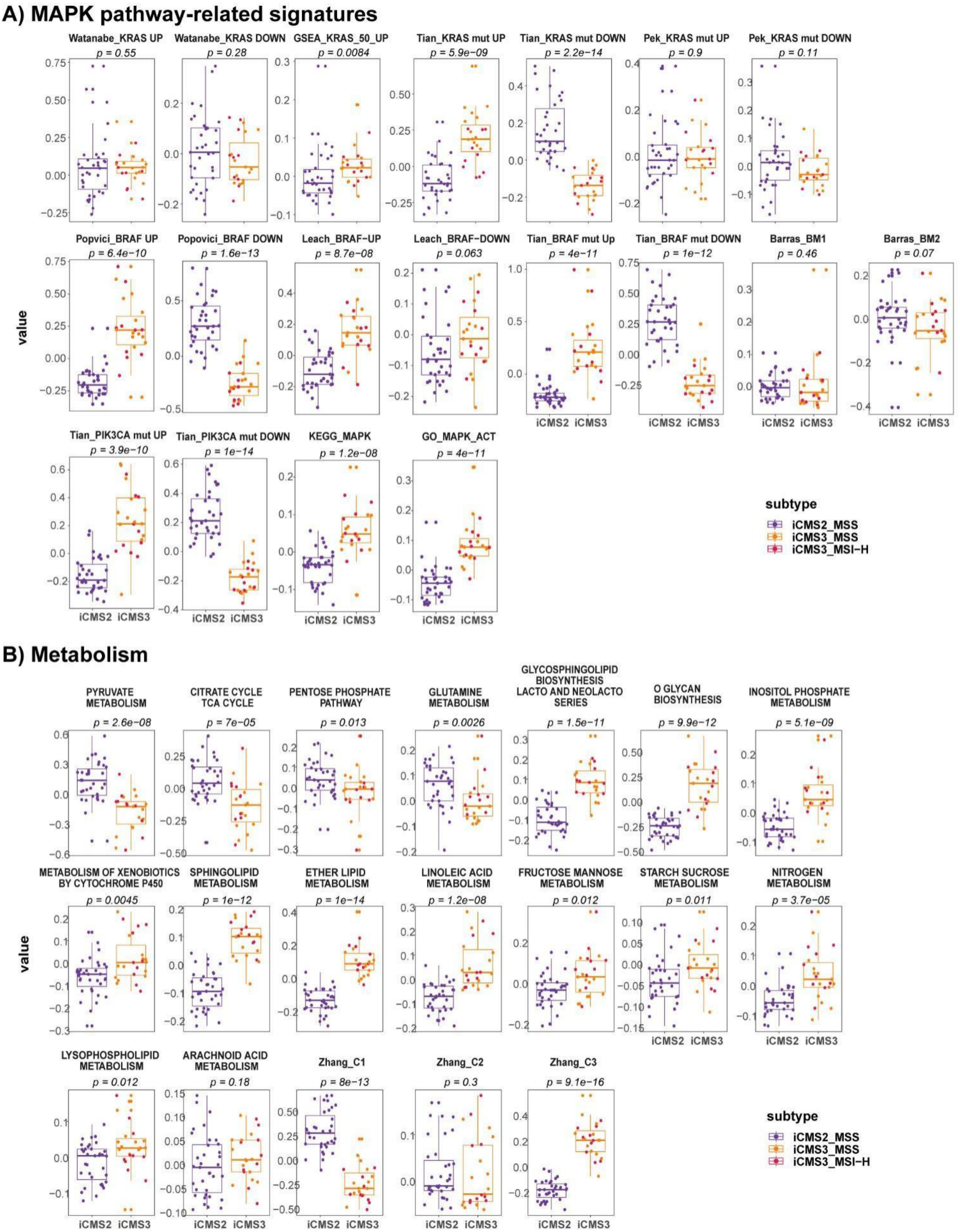

Supplementary Figure 12: Comparison of metagene signature of selected metabolism-related pathways (A) and mutation signatures related to MAPK pathway (B) in single cell epithelial cells across iCMS classes

Box plots of metagene scores comparing iCMS2 (n=35) versus iCMS3 (n=23) in patient-specific pseudo-bulk. Within the iCMS3 group, i3-MSI (n=10) samples are labelled by red jitter points and i3-MSS samples are labelled by orange jitter points. The metagene scores for each patient pseudo-bulk was calculated by averaging the scaled expressions of all genes in the geneset in the given patient. P-values were calculated by two-sided Wilcoxon rank-sum test. Center line indicate the median, and box edges indicate the 25th (Q1)

and 75th (Q3) percentiles. Whiskers are plotted at 1.5xIQR and data beyond the end of the whisker are outliers.

References

1. Aibar, S. *et al.* SCENIC: single-cell regulatory network inference and clustering. *Nat. Methods* 14, 1083–1086 (2017).
2. Hao, Y. *et al.* Integrated analysis of multimodal single-cell data. *Cell* 184, 3573–3587.e29 (2021).
3. Pelka, K. *et al.* Spatially organized multicellular immune hubs in human colorectal cancer. *Cell* 184, 4734–4752.e20 (2021).
4. Hoshida, Y. Nearest template prediction: a single-sample-based flexible class prediction with confidence assessment. *PLoS One* 5, e15543 (2010).
5. Guinney, J. *et al.* The consensus molecular subtypes of colorectal cancer. *Nat. Med.* 21, 1350–1356 (2015).
6. Liu, J. *et al.* An Integrated TCGA Pan-Cancer Clinical Data Resource to Drive High-Quality Survival Outcome Analytics. *Cell* 173, 400–416.e11 (2018).
7. Subramanian, A. *et al.* Gene set enrichment analysis: a knowledge-based approach for interpreting genome-wide expression profiles. *Proc. Natl. Acad. Sci. U. S. A.* 102, 15545–15550 (2005).
8. Love, M. I., Huber, W. & Anders, S. Moderated estimation of fold change and dispersion for RNA-seq data with DESeq2. *Genome Biol.* 15, 550 (2014).
9. Browaeys, R., Saelens, W. & Saeys, Y. NicheNet: modeling intercellular communication by linking ligands to target genes. *Nat. Methods* 17, 159–162 (2020).
10. Hou, R., Denisenko, E., Ong, H. T., Ramilowski, J. A. & Forrest, A. R. R. Predicting cell-to-cell communication networks using NATMI. *Nat. Commun.* 11, 5011 (2020).
11. Chen, B. *et al.* Human colorectal pre-cancer atlas identifies distinct molecular programs underlying two major subclasses of pre-malignant tumors. *bioRxiv* 2021.01.11.426044 (2021) doi:10.1101/2021.01.11.426044.
12. Jain, A. & Tuteja, G. TissueEnrich: Tissue-specific gene enrichment analysis. *Bioinformatics* vol. 35 1966–1967 (2019).

13. Uhlén, M. *et al.* Proteomics. Tissue-based map of the human proteome. *Science* 347, 1260419 (2015).
14. Null, N. *et al.* The Genotype-Tissue Expression (GTEx) pilot analysis: Multitissue gene regulation in humans. *Science* 348, 648–660 (2015).
15. Shen, Y. *et al.* A map of the cis-regulatory sequences in the mouse genome. *Nature* 488, 116–120 (2012).
16. Rees, M. G. *et al.* Correlating chemical sensitivity and basal gene expression reveals mechanism of action. *Nat. Chem. Biol.* 12, 109–116 (2016).
17. Yang, W. *et al.* Genomics of Drug Sensitivity in Cancer (GDSC): a resource for therapeutic biomarker discovery in cancer cells. *Nucleic Acids Res.* 41, D955–61 (2013).
