# Supplementary material for: Single-cell and bulk transcriptome sequencing identifies two epithelial tumor cell states and refines the consensus molecular classification of colorectal cancer
Source: Nat Genet. 2022 Jun 30;54(7):963–75. doi: 10.1038/s41588-022-01100-4 (PMC9279158; doi:10.1038/s41588-022-01100-4)
Supplement: Supplementary file 2 — Reporting Summary [file 41588_2022_1100_MOESM2_ESM.pdf]

nature portfolio

Corresponding author(s): Iain Beehuat Tan

Last updated by author(s): May 10, 2022

Reporting Summary

Nature Portfolio wishes to improve the reproducibility of the work that we publish. This form provides structure for consistency and transparency in reporting. For further information on Nature Portfolio policies, see our Editorial Policies and the Editorial Policy Checklist.

Statistics

For all statistical analyses, confirm that the following items are present in the figure legend, table legend, main text, or Methods section.

n/a	Confirmed	
☐	☒	The exact sample size (*n*) for each experimental group/condition, given as a discrete number and unit of measurement
☒	☐	A statement on whether measurements were taken from distinct samples or whether the same sample was measured repeatedly
☐	☒	The statistical test(s) used AND whether they are one- or two-sided<br>*Only common tests should be described solely by name; describe more complex techniques in the Methods section.*
☐	☒	A description of all covariates tested
☐	☒	A description of any assumptions or corrections, such as tests of normality and adjustment for multiple comparisons
☐	☒	A full description of the statistical parameters including central tendency (e.g. means) or other basic estimates (e.g. regression coefficient) AND variation (e.g. standard deviation) or associated estimates of uncertainty (e.g. confidence intervals)
☐	☒	For null hypothesis testing, the test statistic (e.g. *F*, *t*, *r*) with confidence intervals, effect sizes, degrees of freedom and *P* value noted<br>*Give P values as exact values whenever suitable.*
☒	☐	For Bayesian analysis, information on the choice of priors and Markov chain Monte Carlo settings
☒	☐	For hierarchical and complex designs, identification of the appropriate level for tests and full reporting of outcomes
☐	☒	Estimates of effect sizes (e.g. Cohen's *d*, Pearson's *r*), indicating how they were calculated

Our web collection on statistics for biologists contains articles on many of the points above.

Software and code

Policy information about availability of computer code

Data collection	No specialized software was used for data acquisition
Data analysis	All software used in this study are published and cited either in the main text or Online method. Here is the list of software used in this study: Burrows–Wheeler Aligner (v.0.7.17), Genome Analysis Toolkit 4 (GATK4, v.4.0.2.1), CellRanger version 3.1, RCAv2 (2.0.0), DoubletFinder version (2.0.3), DUBStepR (1.2.0), Seurat (4.0.4), CMSclassifier (1.0.0), inferCNV (1.7.1), DESeq2 (1.30), pySCENIC (0.10.3), GRNboost2 (arboreto 0.1.5), CMScaller (2.0.1), nclust (2.2.3), survival (3.2-7), GSEA software (4.1.0), Complexheatmap (2.6.2), NicheNet (1.0.0), NATMI (git commit: 3ef1f05), TissueEnrich (1.10.1)

For manuscripts utilizing custom algorithms or software that are central to the research but not yet described in published literature, software must be made available to editors and reviewers. We strongly encourage code deposition in a community repository (e.g. GitHub). See the Nature Portfolio guidelines for submitting code & software for further information.

Data

Policy information about availability of data

All manuscripts must include a data availability statement. This statement should provide the following information, where applicable:

- Accession codes, unique identifiers, or web links for publicly available datasets
- A description of any restrictions on data availability
- For clinical datasets or third party data, please ensure that the statement adheres to our policy

The raw scRNA-seq data are available in the European Genome-phenome Archive (EGA) database with accession of EGAD00001008555 (CRC-SG1 cohort), EGAD00001008584 (new KUL3 cohort), and EGAD00001008585 (KUL5 cohort). The raw bulk RNA-seq, whole exome sequencing (WES), and whole genome sequencing (WGS) of Singapore colorectal cancer patients (SG-BULK) are available in EGA database with accession codes: EGAD00001008512 (bulk RNA-seq);

EGAD00001008543 (exome sequencing); EGAD00001008566, EGAD00001008574, EGAD00001008592, EGAD00001008625, and EGAD00001008637 (whole-genome sequencing). Processed TPM (SG-BULK) and count expression matrices (scRNA-seq from 5 cohorts) are available through Synapse under the accession codes syn26720761 and syn26844071, respectively. Published raw scRNA-seq data referenced in the study are available from EGA under the accession codes EGAS00001003779 and EGAS00001003769 (SMC cohort) and from ArrayExpress under the accession codes E-MTAB-8410 and E-MTAB-8412 (KUL3 cohort).

Field-specific reporting

Please select the one below that is the best fit for your research. If you are not sure, read the appropriate sections before making your selection.

☒ Life sciences ☐ Behavioural & social sciences ☐ Ecological, evolutionary & environmental sciences

For a reference copy of the document with all sections, see nature.com/documents/nr-reporting-summary-flat.pdf

Life sciences study design

All studies must disclose on these points even when the disclosure is negative.

Sample size	The sample size of 63 represents 1 of the largest single cell datasets to date and was the largest, that we were aware of at the point of study conception. We secured budget to be able to perform analyses that represent a 10 fold increase in cell numbers compared to other colorectal cancer studies at the point of study data generation. This study design would enable a diversity of cells comparable in scale to current studies being published in leading journals.
Data exclusions	There is no specific data exclusion.
Replication	Our study has findings that have been replicated across 5 cohorts internally with 63 patients and nearly half a million cells. We then replicated our study in a recent study of 62 patients also with around half a million cells.
Randomization	Randomization is not applicable as this is not a therapeutic trial.
Blinding	The researchers were blind to the clinical annotations at the point of biologic discovery.

Reporting for specific materials, systems and methods

We require information from authors about some types of materials, experimental systems and methods used in many studies. Here, indicate whether each material, system or method listed is relevant to your study. If you are not sure if a list item applies to your research, read the appropriate section before selecting a response.

Materials & experimental systems

n/a	Involved in the study	
☒	☐	Antibodies
☒	☐	Eukaryotic cell lines
☒	☐	Palaeontology and archaeology
☒	☐	Animals and other organisms
☐	☒	Human research participants
☒	☐	Clinical data
☒	☐	Dual use research of concern

Methods

n/a	Involved in the study	
☒	☐	ChIP-seq
☒	☐	Flow cytometry
☒	☐	MRI-based neuroimaging

Human research participants

Policy information about studies involving human research participants

Population characteristics	Patients with colorectal cancer undergoing primary surgery for colorectal cancer at National Cancer Centre Singapore, Singapore General Hospital, Samsung cancer centre, University Leuven. We have provided individual level data of the gender and age and other clinical metadata in the supplementary tables that accompany the manuscript
Recruitment	Patients undergoing primary surgery for colorectal cancer at National Cancer Centre Singapore, Singapore General Hospital, Samsung cancer centre, University Leuven were approached for consent to donate research tissue for research. Patients had to have sufficient tumor volume to be able to support collection of extra tissue beyond what would be required for pathologic diagnosis. At surgical removal, a research pathologist evaluates if there is sufficient tissue that can be collected for research use without compromising diagnostic staging.<br><br>We recruited patients whose tumors were of sufficient size to enable sufficient collection of tumor aliquots that are to be dissociated for single cell analyses. This may have led to a selection bias towards tumors that are of sufficient size to enable analyses. This is inherent in single cell studies. The mapping of the data to bulk transcriptomics data as validation, where this effect of tumor size is no longer there, suggest our findings are unlikely to be affected by this minimum (2cm) size requirement.

Ethics oversight	The study was approved by the institutional review boards of Singhealth (2018-2795 and 2018-2376) for CRC-SG1 & CRC-SG2, Samsung Medical Center (approval no. SMC2017-07-131) for the SMC and Commissie Medische Ethiek UZ KU Leuven/ Onderzoek (approval no. S50887-ML4707) for the KUL3 and KUL5 datasets, respectively. All mentioned datasets/studies were carried out in accordance with ethical guidelines and all patients provided written informed consent.

Note that full information on the approval of the study protocol must also be provided in the manuscript.
